# An Isolable Three‐Coordinate Germanone and Its Reactivity

**DOI:** 10.1002/chem.202102972

**Published:** 2021-10-05

**Authors:** Xuan‐Xuan Zhao, Tibor Szilvási, Franziska Hanusch, Shigeyoshi Inoue

**Affiliations:** ^1^ Department of Chemistry WACKER-Institute of Silicon Chemistry and Catalysis Research Center Technische Universität München Lichtenbergstraße 4 85748 Garching bei München Germany; ^2^ Department of Chemical and Biological Engineering University of Alabama Tuscaloosa AL 35487 USA

**Keywords:** carbonyls, germanone, *N*-heterocyclic imines, O-atom transfer, small molecule activation

## Abstract

A rare three‐coordinate germanone [IPrN]_2_Ge=O (IPrN=bis(2,6‐diisopropylphenyl)imidazolin‐2‐imino) was successfully isolated. The germanone has a rather high thermal stability in arene solvent, and no detectable change was observed at 80 °C for at least one week. However, high thermal stability of [IPrN]_2_Ge=O does not prevent its reactivity toward small molecules. Structural analysis and initial reactivity studies revealed the highly polarized nature of the terminal Ge=O bond. Besides, the addition of phenylacetylene, as well as O‐atom transfer with 2,6‐dimethylphenyl isocyanide make it a mimic of nucleophilic transition‐metal oxides. Mechanism for O‐atom transfer reaction was investigated via DFT calculations, which revealed that the reaction proceeds via a [2+2] cycloaddition intermediate.

## Graphical abstract

**A rare three‐coordinate germanone**: bis(imino)germanone [IPrN]_2_Ge=O (IPrN=bis(2,6‐diisopropylphenyl)imidazolin‐2‐imino) was successfully isolated. Initial reactivity studies combined with experimental and computational bonding analysis revealed highly polarized nature of the terminal Ge=O bond. Moreover, transition metal oxide‐like reactivity has been observed for the addition of phenylacetylene, as well as O‐atom transfer with 2,6‐dimethylphenyl isocyanide.

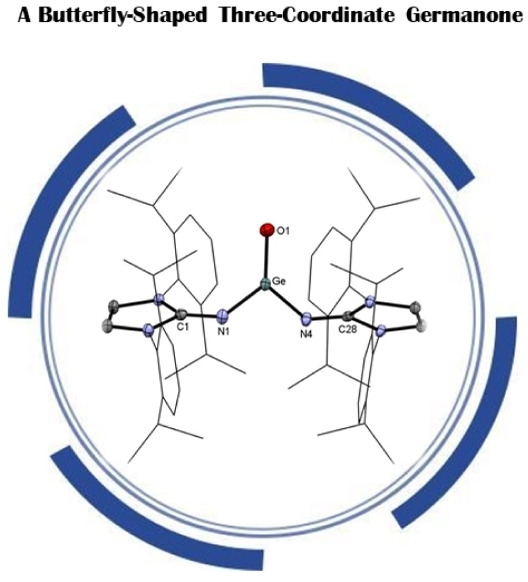

Whereas carbonyl compounds are irreplaceable and highly versatile building blocks in today's organic synthesis, their heavier analogues (R_2_E=O, E=group 14 element), are still rare and have been much less explored. Specifically germanones (R_2_Ge=O), were long thought to be elusive and unstable intermediates,^[1]^ until the first evidence of organogermanium oxides was reported by Satgé in 1971.^[2]^ The high reactivity stems from the unfavorable *pπ‐pπ* overlap between oxygen and electropositive germanium atoms, that results in weak and polarized Ge−O bonds.^[3]^ Thermodynamic and kinetic stabilization was utilized to prevent their oligomerization/polymerization,^[3g]^ thus affording several milestones in germanone chemistry. Besides the stable heavier ketones R_2_Ge=X with a terminal heavier group 16 element (X=S, Se, or Te),^[4]^ the isolation of several donor‐acceptor‐ or solely donor‐stabilized Ge=O complexes has been achieved by employing additional Lewis acids or bases.^[5]^ Following this strategy, the seminal breakthrough towards tetra‐coordinate germanones **I** was described by Driess in 2009 (Scheme 1). Coordination of NHC or 4‐*N*,*N*‐dimethylaminopyridine (DMAP) results in a distorted tetrahedral geometry around the electron‐deficient germanium center.[^5a^, ^5b^]

**Scheme 1:**
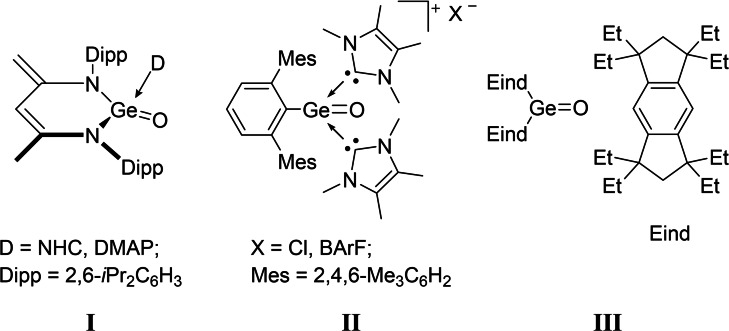
Selected examples of germanones, as well as the NHC‐stabilized germa‐acylium Ion II.

More recently, we have shown the successful isolation of the NHC‐stabilized germa‐acylium ion **II**, which was utilized in catalytic CO_2_ functionalizations (Scheme 1).^[6]^ In 2012, Tamao, Matsuo and coworkers reported the first isolation of the “genuine” germanone **III** with a three‐coordinate germanium atom multiply bonded to oxygen, which is stabilized by the rigid and bulky Eind groups (Eind=1,1,3,3,5,5,7,7‐octaethyl‐*s*‐hydrindacen‐4‐yl; Scheme 1).^[7a]^ The landmark discovery opened the door for the chemistry of heavier group 14 carbonyls.^[8]^ Whilst significant advances have been made, the isolation of acid‐base free germanones still remains challenging.

In 2017, we successfully isolated the first stable neutral acyclic three‐coordinate silanones by combining *π*‐donating *N*‐heterocyclic imino (NHI) and *σ*‐donating silyl groups.^[8e]^ Moreover, in 2018, the group of Dielmann reported two NHI supported Lewis base free oxophosphonium monocations, which represent the first example of a phosphacarbonyl species.^[9]^ Motivated by these results, we set out to stabilize the polarized Ge=O moiety by using two NHI ligands.^[10]^ Accordingly, we found that the bis(imino)germylene,^[11]^ reported by the group of Rivard, could be an ideal precursor for our targeted three‐coordinate germanones. Herein we disclose the isolation, structural characterization, and initial reactivity study of a three‐coordinate germanone with two NHI ligands.

The bis(imino)germylene **1** was synthesized in a modified literature‐known procedure.^[11]^ Reaction of GeCl_2_•dioxane with two equivalents of IPrNLi (IPrN=bis(2,6‐diisopropylphenyl)imidazolin‐2‐imino) in dry THF gave **1** in high yield (88 %). Indeed, treatment of a toluene solution of **1** with gaseous N_2_O (1.0 bar) at room temperature led to the desired product **2** as an orange solid (89 %; Scheme 2). The bis(imino)germanone **2** has a rather high thermal stability in arene solvent; no detectable change was observed in the ^1^H NMR spectrum of **2** in C_6_D_6_ at 80 °C for at least one week. The characteristic Ge=O stretching vibration was found at 912 cm^−1^ in the IR spectrum (calc. 907 cm^−1^), which is comparable to the reported Ge=O stretching in Eind_2_Ge=O (**III**; 916 cm^−1^).

**Scheme 2:**
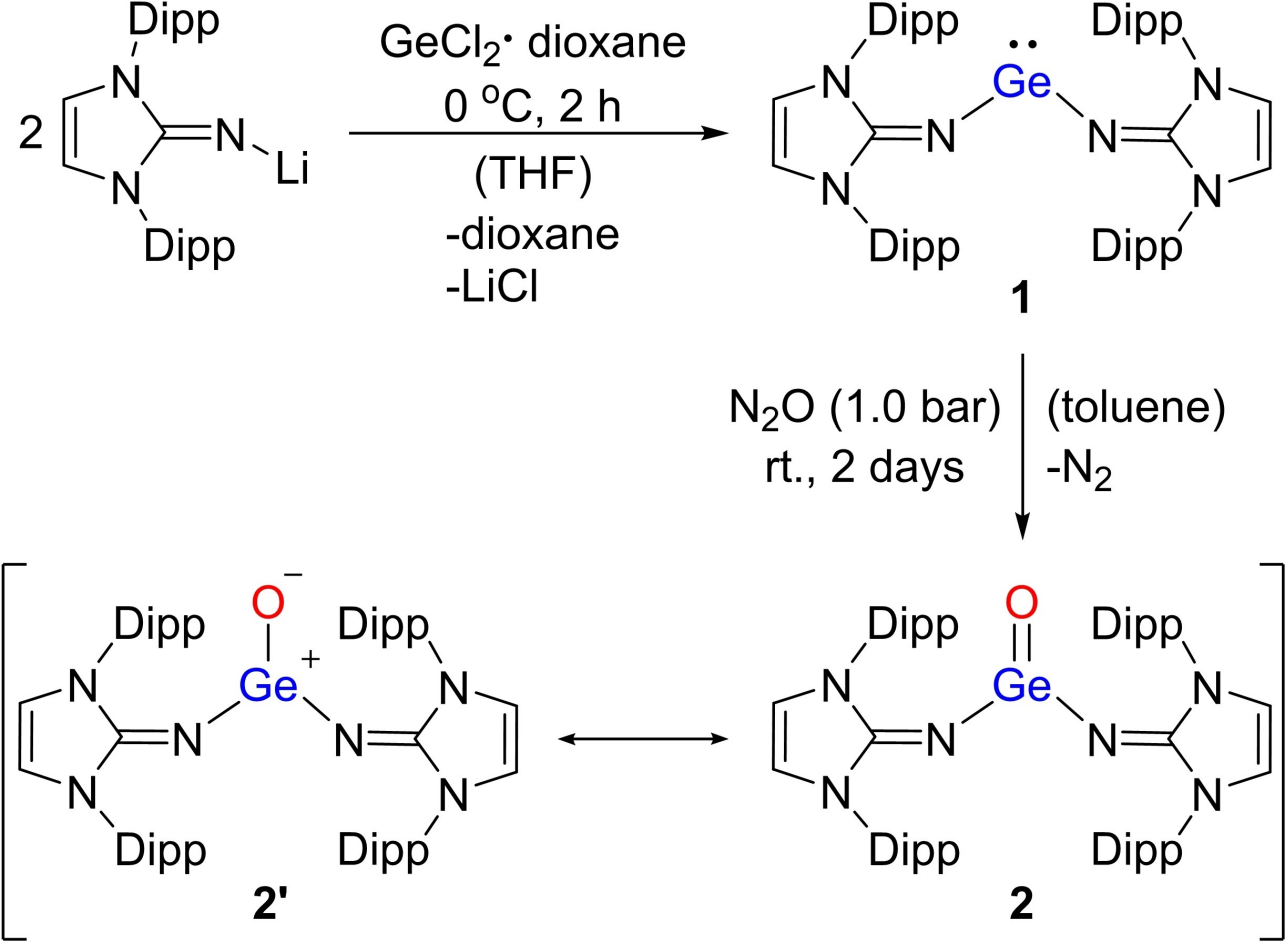
Synthesis of bis(imino)germanone **2** from bis(imino)germylene **1**.

Pale‐yellow crystals of **2** were obtained from a saturated solution in Et_2_O at −30 °C. Single crystal X‐ray diffraction (SC‐XRD) analysis unambiguously confirmed the monomeric structure of **2** in the solid state (Figure 1a).^[12]^ In addition, the Ge=O moiety in **2** lies within a protecting pocket formed by the flanking shielding ligands (Figure 1b). The molecular structure revealed a trigonal planar geometry at the germanium center (sum of bonding angles: 360°) and the Ge1−O1 bond length of **2** (1.6494(10) Å), which is almost identical to that in Eind_2_Ge=O (**III**; 1.6468(5) Å), and generally shorter than that in base‐stabilized tetra‐coordinate germanones (1.664–1.718 Å).^[5]^ The Ge−N bonds (Ge1−N1 1.7819(12) Å / Ge1−N4 1.7825(12) Å) are shortened and neighboring N−C bonds (N1−C1 1.2872(18) Å / N4−C28 1.2914(18) Å) are elongated, compared to that in precursor **1** (Ge−N: 1.8194(15) Å; N−C: 1.273(2) Å), thus suggesting admixture of a partial metalimide character in the neutral complex **2**.^[10]^

**Figure 1:**
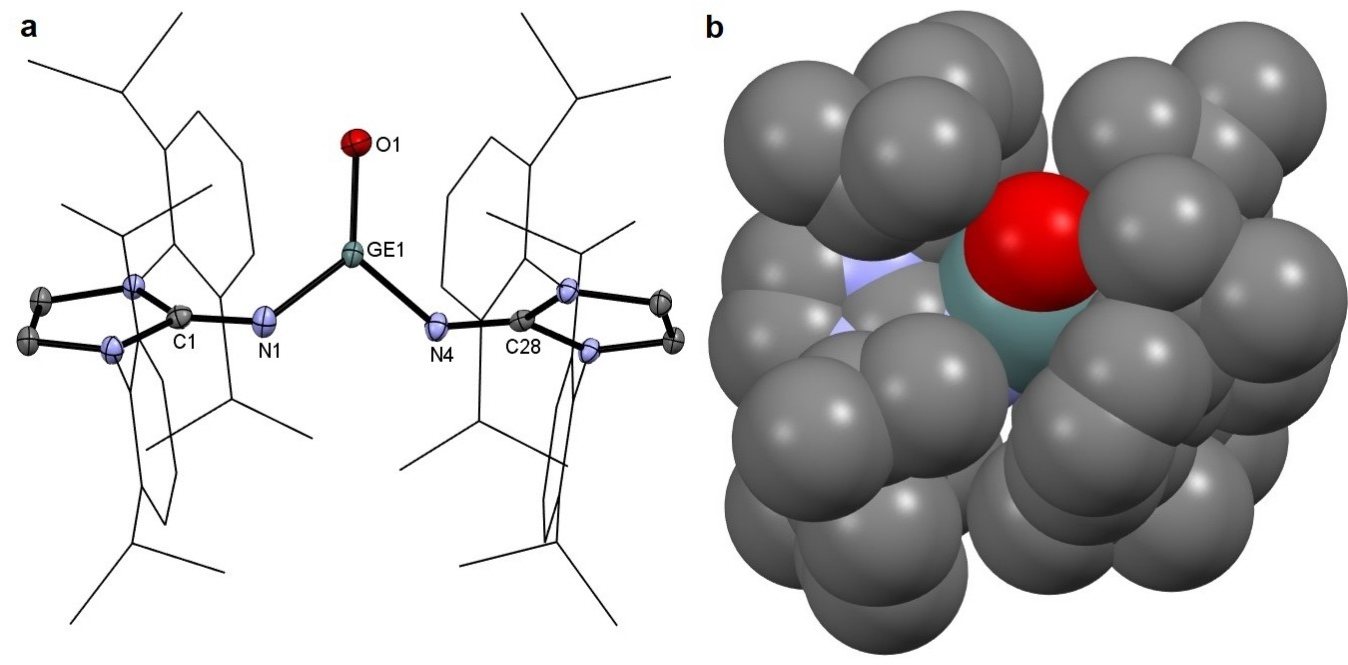
Molecular structure (a) and space filling representation (b) of **2**. Thermal ellipsoids are shown at 50 % probability level. Hydrogen atoms are omitted for clarity. Selected bond lengths [Å] and angles [°]: Ge1−O1 1.6494(10), Ge1−N1 1.7819(12), Ge1−N4 1.7825(12), N1−C1 1.2872(18), N4−C28 1.2914(18), O1−Ge1−N1 125.94(6), O1−Ge1−N4 125.41(6), N1−Ge1−N4 108.65(6), Ge1−N1−C1 127.81(10), Ge1−N4−C28 125.02(10).

Density Functional Theory (DFT) calculations were performed to understand the electronic structure of **2**. We found that the Wiberg Bond Index (WBI; Table S3) indicates partial double bond character for Ge=O (1.30). Natural Population Analysis showed a positive Ge center (+1.89; Table S3) and a negatively charged O center (−1.02), whereas Natural Bond Orbital analysis shows only one Ge−O bond (Table S2). Analyzing the molecular orbitals (Figure S35) shows that the HOMO is mainly located on the *π*‐system of the IPrN groups, while the LUMO is associated with the *π*‐system of the phenyl rings of **2**. HOMO−3 and LUMO+9 are the orbitals that are directly associated with the Ge=O *π*‐bonding (Figure S35), which depict an O‐dominated *π*‐orbital and a Ge‐dominated π*‐orbital, respectively. Interestingly, HOMO−3 and LUMO+9 indicate very little coupling to the N atoms of the IPrN groups, which also suggests that Ge=O can be described as a double bond although the zwitterionic resonance structure **2’** (Scheme 2) should not be neglected based on the other computational metrics.

The polarized Ge=O bond of **2** is reflected by the following reactivity study (Scheme 3 and 4). The products in all cases were identified by multinuclear and 2D NMR spectroscopy, and elemental analysis (EA; see Supporting Information for details). Germanone **2** shows reactivity toward pinacolborane (HBpin), bromotrimethylsilane (TMSBr) and phenylsilane (PhSiH_3_), with polarized B−H, Si−Br or Si−H single bonds, to immediately afford the corresponding 1,2‐adducts **3**, **4** and **5** at room temperature. The ^1^H NMR signal of the Ge−H bond in **3** appears at 5.04 ppm, which is similar to that in **5** (4.78 ppm).

**Scheme 3:**
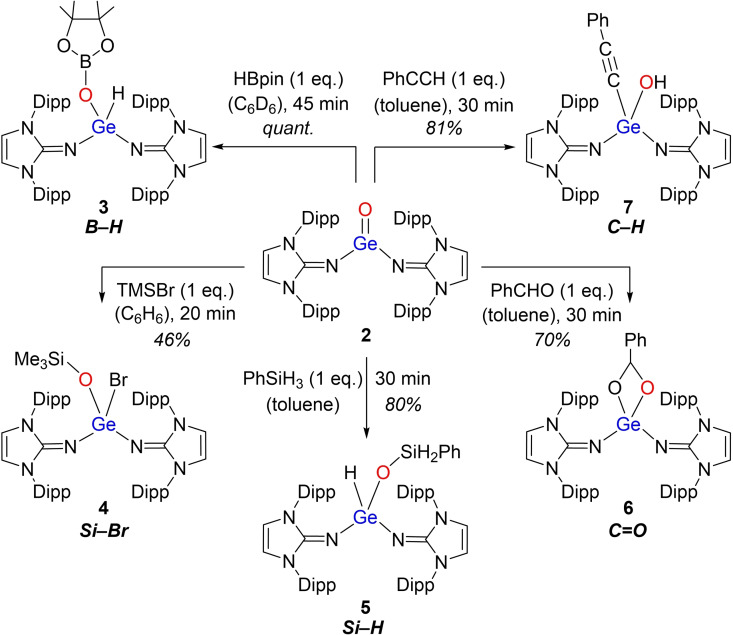
Reactivity of bis(imino)germanone **2**.

**Scheme 4:**
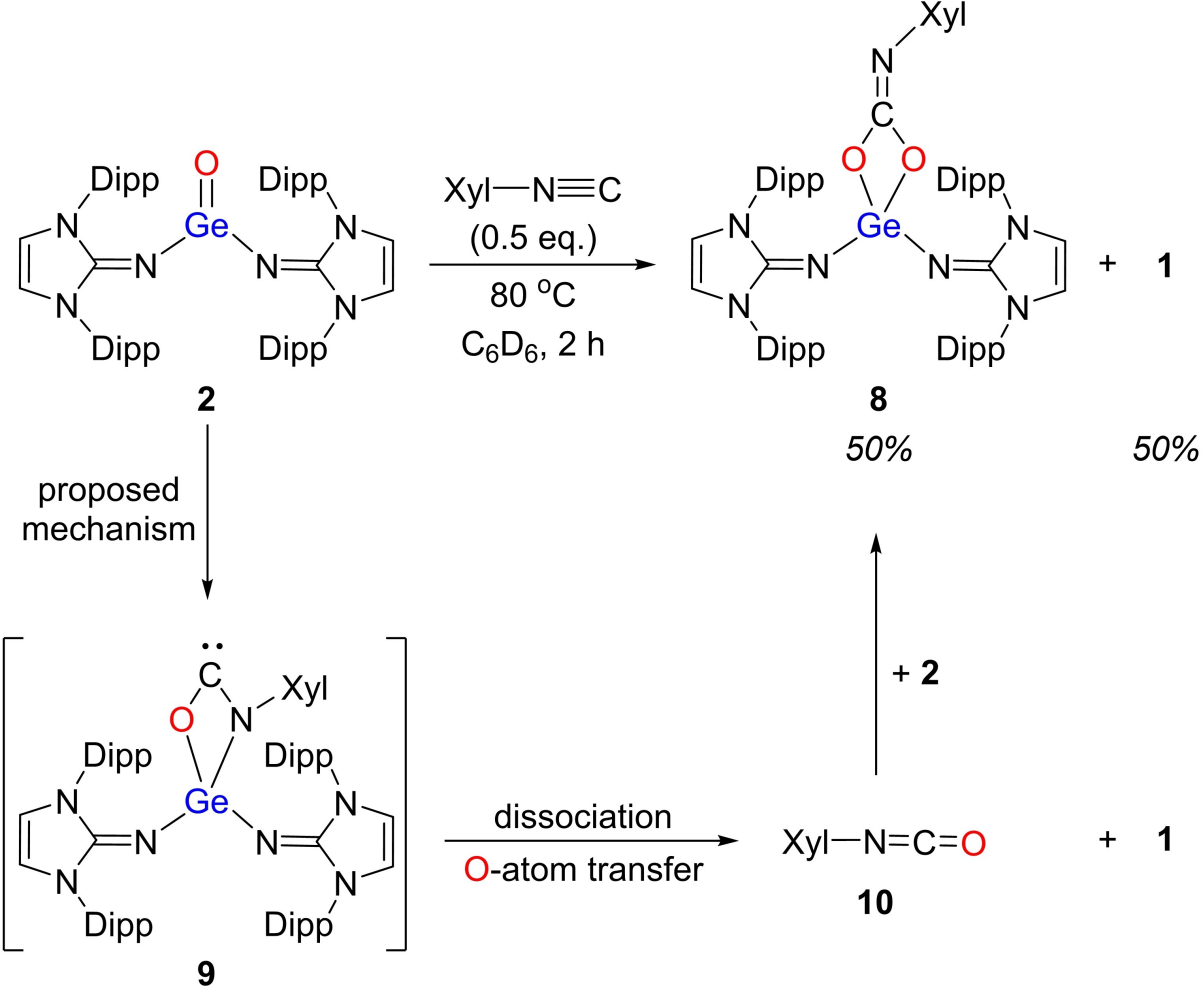
Reaction of bis(imino)germanone **2** with 2,6‐dimethylphenyl isocyanide (CNXyl).

Compared to the analog PhSiH_3_ reaction product of **III** (Eind_2_Ge(H)OSiH_2_Ph; Ge−H: 7.94 ppm), the Ge−H signal in **5** is significantly upfield shifted, which could be attributed to the strongly *π*‐electron donating NHI ligands. In consequence, full conversion of **2** to **5** was observed after 30 min, whereas **III** was reacted for one day with PhSiH_3_ until completion of the reaction could be confirmed.^[7a]^

Moreover, **2** reacted with the C=O bond of benzaldehyde (PhCHO), to smoothly furnish the cyclic product **6**. The formation of the four‐membered heterocycle in **6** was confirmed by ^1^H/^13^C HSQC and HMBC NMR spectroscopy, revealing a singlet at 99.7 ppm for the ring carbon atom, and a singlet at 5.10 ppm for the ring proton.

Since the formation of products **3** to **6** can be attributed to the highly polarized Ge^δ+^−O^δ−^ bond, it has been the scope to test the reactivity of **2** towards other small molecules. Treatment of **2** with H_2_ and NH_3_ showed no conversion and reaction with CO_2_ formed an unidentified product mixture. Upon exposure towards MeOH, germanone **2** was directly converted into the imine IPrNH as a result of high proton affinity of the imidazolin‐2‐iminato ligand. However, the reaction of **2** with a terminal alkyne (phenylacetylene, PhCCH) at room temperature resulted in direct conversion to the hydroxoacetylide complex **7** in good yield (81 %; Scheme 3). The acetylide complex **7**, identified by 2D NMR spectroscopy, shows a sharp OH signal in the ^1^H NMR spectrum at −0.77 ppm (C_6_D_6_). This reactivity is reminiscent of pyridine‐stabilized Ti(IV) oxo complex Cp*_2_(pyridine)Ti=O (Cp*=*η* ^5^‐C_5_Me_5_) reported by Bergman and coworkers.^[13]^

More interestingly, reaction of **2** with 0.5 equivalent of 2,6‐dimethylphenyl isocyanide (CNXyl) led to a mixture of the [2+2] cycloaddition product **8** (50 % NMR yield) and the O‐atom transfer product **1** (50 % NMR yield; Scheme 4). Several attempts to separate product **8** from the reaction mixture remained unsuccessful. To clarify the mechanism, we performed the reaction of **2** with commercially available **10** (1 equiv.) at room temperature, which immediately resulted in the desired compound **8** in nearly quantitative yield.

Computational analysis using DFT was carried out to understand the reactivity of **2** with CNXyl (Figure S34). We found that the [2+2] cycloaddition product **9** is an energetically favored intermediate (−15.4 kcal/mol), while the dissociation and formal O‐atom transfer leads to **10** (−28.0 kcal/mol), which can react with another molecule of **2** to provide the thermodynamically stable product **8** (−52.3 kcal/mol). We note that the calculated high barriers (20.5 kcal/mol and 22.5 kcal/mol) are in general agreement with the observed very slow reaction at room temperature and reaction mechanism can explain the observed mixture of products (**1**+**8**). We also studied other possible pathways but all attempts to locate the direct coordination of CNXyl to Ge and a GeOC three membered ring intermediate with an exocyclic=NXyl unit were not successful. Additionally, we found a direct O‐atom transfer mechanism, but it was less favorable than the cycloaddition pathway (26.0 kcal/mol; Figure S34).

For comparison, the reactivity of a rhenium(III) terminal oxo complex, (*η* ^2^‐DHF)(BDI)Re=O, (DHF=dihydrofulvalene; BDI=*N*,*N’*‐bis(2,6‐diisopropylphenyl)‐2,4‐dimethyl‐*β*‐diketiminate) with isocyanides, R−NC (R=^*t*^Bu, 2,6‐xylyl) was described in 2018.^[14]^ This is suggested to be initiated by the nucleophilic character of the rhenium oxo moiety. Moreover, similar reactions of (Tbt)(Tip)Ge=X (X=S, Se; Tbt=2,4,6‐tris[bis(trimethylsilyl)methyl]phenyl; Tip=2,4,6‐triisopropylphenyl) and PhN=C=S have been reported by Tokitoh.^[3d]^ Therefore, in this reaction, germanone **2** acted not only as a heavy ketone, but also as a mimic of nucleophilic transition metal oxides (TMO). In fact, the O‐atom transfer reaction with isocyanides is prototypical for TMO.

To clarify the reaction mechanism of O‐atom transfer by aiming at the isolation of isocyanide complexes similar to known silicon derivatives,^[15]^ we conducted the reaction of **1** with CNXyl. Surprisingly, **1** does not react with CNXyl (1 equiv.) even at elevated temperatures.

In summary, we have achieved the synthesis and isolation of bis(imino)germanone **2** with a trigonal planar geometry. Thanks to the efficient stabilization by two bulky and strongly *π*‐donating NHI substituents, germanone **2** is remarkable stable in arene solvent for at least one week. High stability makes it easier to handle and allows us to investigate its reactivity towards various molecules. The addition reactions of **2** with pinacolborane (HBpin), bromotrimethylsilane (TMSBr), phenylsilane (PhSiH_3_), and benzaldehyde (PhCHO) demonstrated polarized Ge^δ+^−O^δ−^ reactivity. In addition, the conversion of phenylacetylene (PhCCH), as well as the O‐atom transfer reaction with 2,6‐dimethylphenyl isocyanide (CNXyl) displayed its transition metal oxide‐like behavior. This similarity may provide new opportunities for main group metal mediated catalytic applications in the future.

## Conflict of interest

The authors declare no conflict of interest.

## Supporting information

As a service to our authors and readers, this journal provides supporting information supplied by the authors. Such materials are peer reviewed and may be re‐organized for online delivery, but are not copy‐edited or typeset. Technical support issues arising from supporting information (other than missing files) should be addressed to the authors.

Supporting Information
