# Supplementary material for: An Isolable Three‐Coordinate Germanone and Its Reactivity
Source: Chemistry. 2021 Oct 5;27(64):15914–7. doi: 10.1002/chem.202102972 (PMC9292218; doi:10.1002/chem.202102972)
Supplement: Supplementary file 1 — Supporting Information [file CHEM-27-15914-s001.pdf]

Chemistry–A European Journal

Supporting Information

An Isolable Three-Coordinate Germanone and Its Reactivity

Xuan-Xuan Zhao, Tibor Szilvási, Franziska Hanusch, and Shigeyoshi Inoue*

Table of Contents

1. Experimental Section S2

2. X-ray Crystallographic Data S25

3. Computational Section S27

4. References S56

1. Experimental Section

General Considerations

All experiments and manipulations were carried out under dry oxygen-free argon atmosphere using standard Schlenk techniques or in a glovebox. All glass junctions were coated with PTFE-based grease Merkel Triboflon III. All the solvents were dried and freshly distilled under Ar atmosphere prior to use by standard techniques. The ^{1}H, $^{13}C\{^{1}H\}$, $^{11}B\{^{1}H\}$ NMR spectra were recorded on Bruker 400 MHz spectrometer. Chemical shifts are referenced to (residual) solvent signals. Abbreviations: s = singlet, br = broadened, d = doublet, t = triplet, m = multiplet. ATRFT-IR spectra were recorded on a Bruker Alpha FT-IR spectrometer (diamond ATR, located inside an argon-filled glovebox) in a range of 400 – 4000 cm^{-1}. Elemental analysis (EA) were conducted with a EURO EA (HEKA tech) instrument equipped with a CHNS combustion analyzer. Unless otherwise stated, all commercially available chemicals were purchased from *abcr GmbH*, *Sigma-Aldrich Chemie GmbH* or *Tokyo Chemical Industry Co., Ltd.*, and used without further purification. Dinitrogen monoxide (N_2O) 5.0 (≥99.999%) was purchased from *Westfalen AG* and used as received. The starting materials IPrNLi (IPrN = bis(2,6-diisopropylphenyl)imidazolin-2-imino)[S1] and $GeCl_2$•dioxane[S2] were prepared according to the literature procedures, respectively.

Synthetic Procedures

Synthesis of bis(imino)germylene 1

$GeCl_2$•dioxane (566 mg, 2.44 mmol, 1.0 eq.) dissolved in THF (40 mL) was added dropwise to a solution of IPrNLi (2.0 g, 4.88 mmol, 2.0 eq.) in THF (60 mL) at 0 °C. The reaction mixture was stirred for 2 h at 0 °C. The volatiles were removed *in vacuo* and the solid residue was extracted with toluene (10 mL × 3). After filtration the solvent was removed from the filtrate *in vacuo* to obtain a yellow solid (1.89 g, 88%).

^{1}H NMR (400 MHz, C_6D_6) δ = 7.25 (t, J = 7.7 Hz, 4H, Ar$\underline{H}$), 7.10 (d, J = 7.7 Hz, 8H, Ar$\underline{H}$), 5.93 (s, 4H, NC$\underline{H}$), 3.07 (septet, J = 6.8 Hz, 8H, C$\underline{H}$(CH_3)$_2$), 1.18 (d, J = 6.8 Hz, 24H, CH(C$\underline{H}_3$)$_2$), 1.15 (d, J = 6.8 Hz, 24H, CH(C$\underline{H}_3$)$_2$).

^{13}C NMR (101 MHz, C_6D_6) δ = 148.6 (N$\underline{C}$N), 148.6 (N$\underline{C}$Ar), 134.6 (Ar$\underline{C}$), 129.1 (Ar$\underline{C}$), 123.8 (Ar$\underline{C}$), 113.9 (N$\underline{C}$H), 28.9 ($\underline{C}$H(CH_3)$_2$), 24.5 (CH($\underline{C}H_3$)$_2$), 23.7 (CH($\underline{C}H_3$)$_2$).

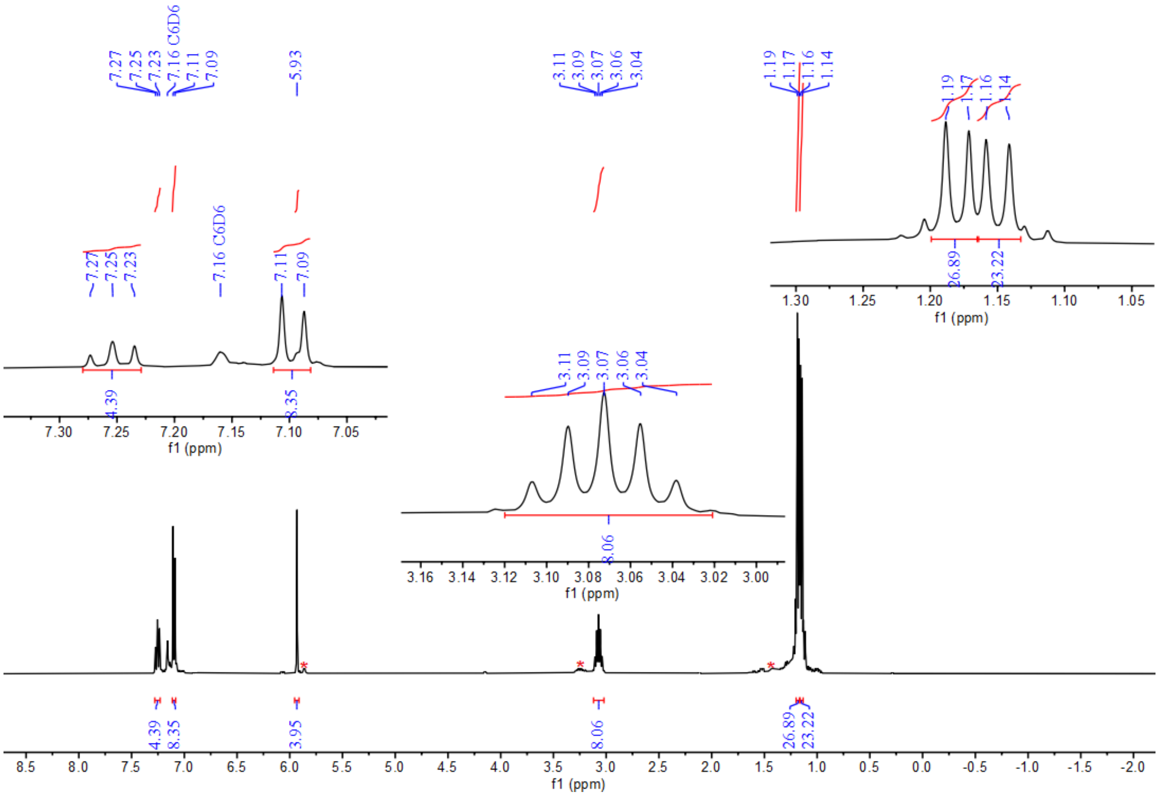

Figure S1. ^{1}H NMR spectrum of bis(imino)germylene **1**. The resonances of IPrNH, resulting from minor complex decomposition in solution, are marked with asterisks *.

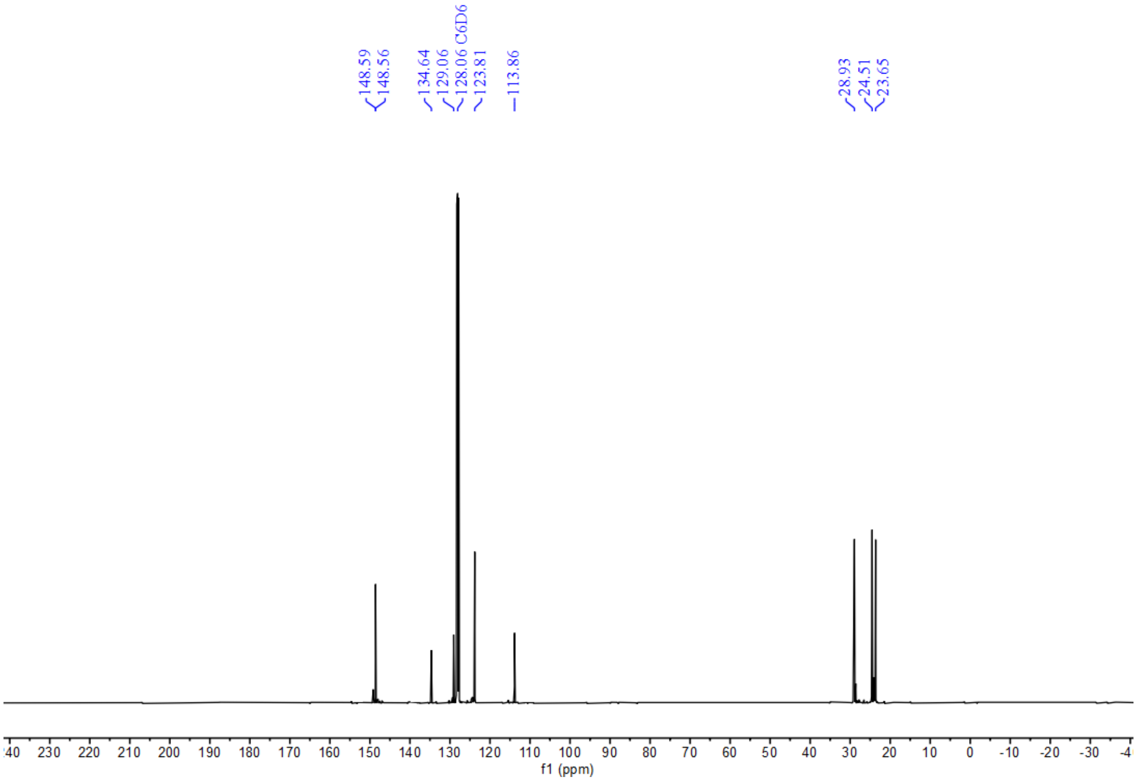

Figure S2. ^{13}C NMR spectrum of bis(imino)germylene **1**.

Synthesis of bis(imino)germanone 2

The solution of **1** (1.14 g, 1.30 mmol) in toluene (20 mL) in a Schlenk tube equipped with a PTFE-coated magnetic stirring bar was cooled to be solidified. The upper argon atmosphere was replaced with N_2O gas (1.0 bar). The reaction mixture was allowed to warm to room temperature, and then was stirred for 2 days. Removal of the solvent gave an orange crude solid, and **2** was recrystallized from a saturated solution in Et_2O at –30 °C as pale-yellow crystals (1.03 g, 89%).

^{1}H NMR (400 MHz, C_6D_6) δ = 7.27 (t, J = 7.7 Hz, 4H, Ar<u>*H*</u>), 7.12 (d, J = 7.7 Hz, 8H, Ar<u>*H*</u>), 5.92 (s, 4H, NC<u>*H*</u>), 2.93 (septet, J = 6.8 Hz, 8H, C<u>*H*</u>(CH_3)$_2$), 1.24 (d, J = 6.9 Hz, 24H, CH(C<u>*H*</u>$_3$)$_2$), 1.12 (d, J = 6.9 Hz, 24H, CH(C<u>*H*</u>$_3$)$_2$).

^{13}C NMR (101 MHz, C_6D_6) δ = 149.5 (N<u>*C*</u>N), 148.2 (N<u>*C*</u>Ar), 133.1 (Ar<u>*C*</u>), 129.8 (Ar<u>*C*</u>), 124.4 (Ar<u>*C*</u>), 114.4 (N<u>*C*</u>H), 29.1 (<u>*C*</u>H(CH_3)$_2$), 24.8 (CH(<u>*C*</u>H$_3$)$_2$), 23.6 (CH(<u>*C*</u>H$_3$)$_2$).

Anal. Calcd. [%] for $C_{54}H_{72}GeN_6O$: C, 72.56; H, 8.12; N, 9.40. Found [%]: C, 72.46; H, 8.23; N, 9.52.

IR: $\nu_{Ge=O}$ = 912 cm^{-1}.

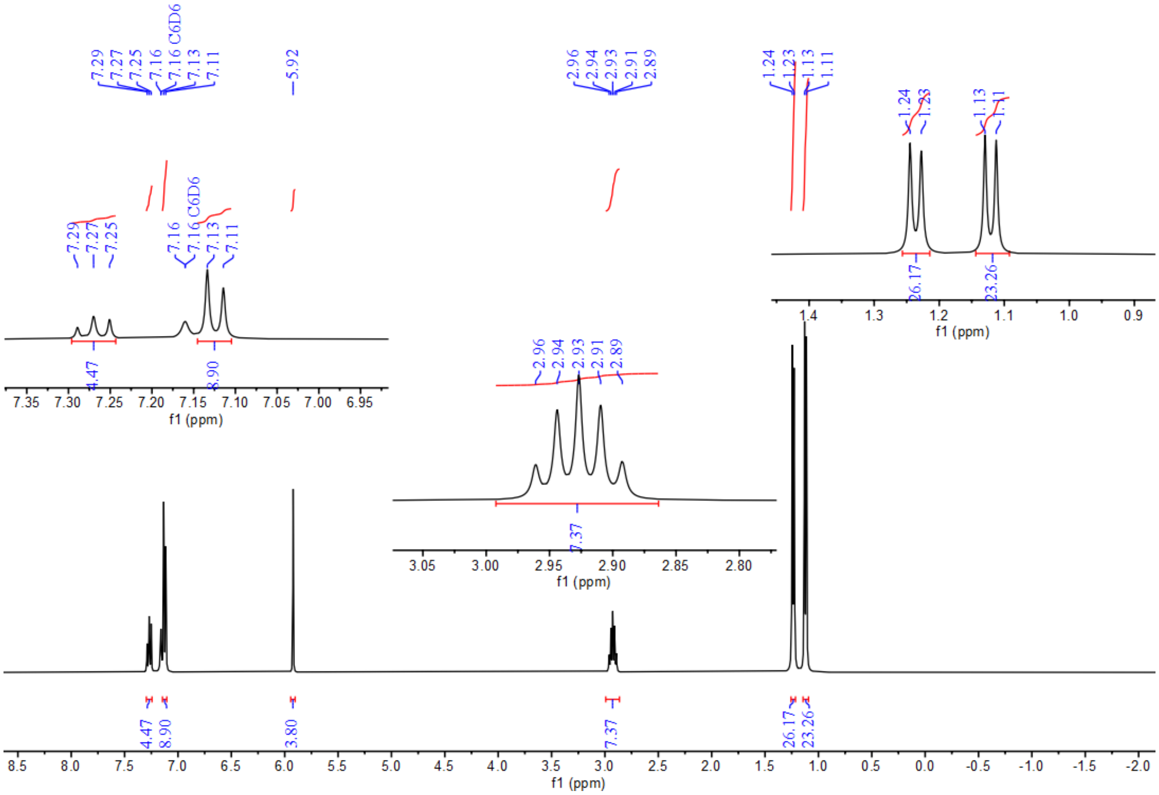

Figure S3. ^{1}H NMR spectrum of bis(imino)germanone **2**.

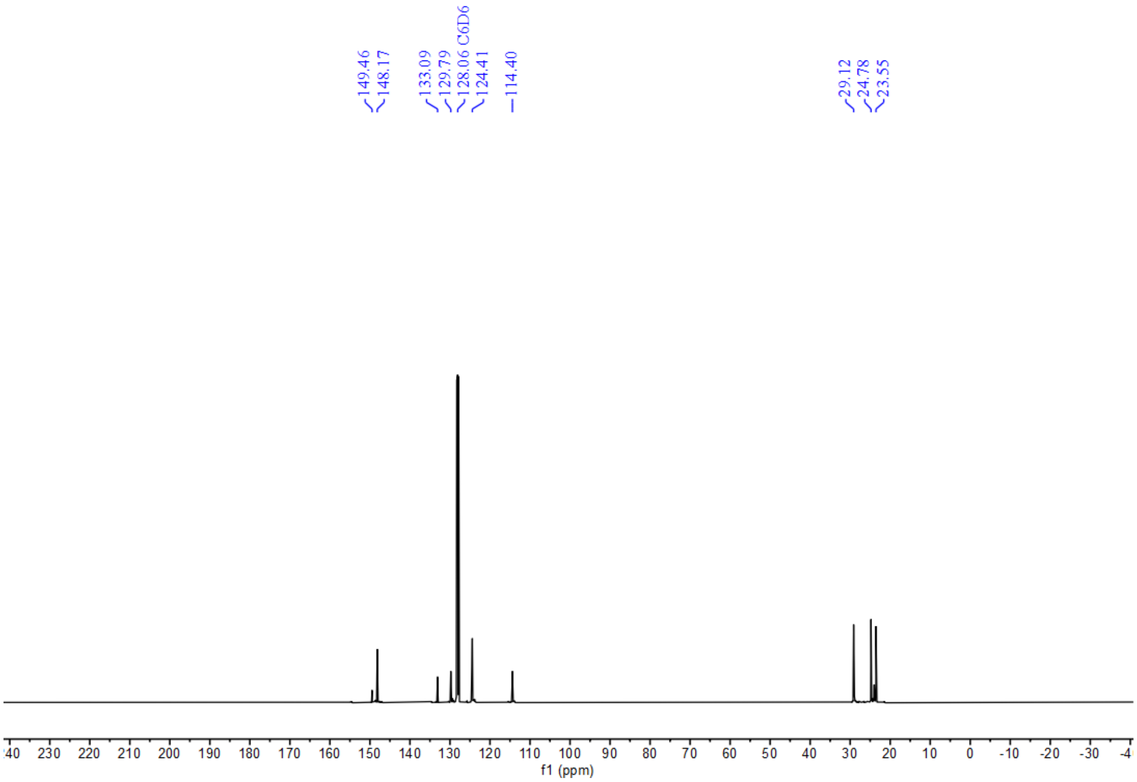

Figure S4. ^{13}C NMR spectrum of bis(imino)germanone **2**.

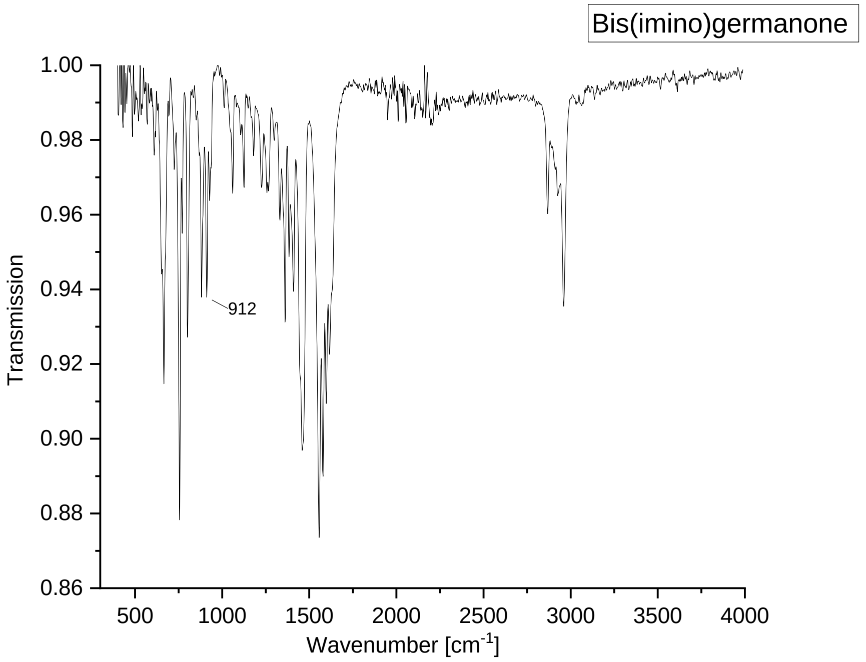

Figure S5. IR spectrum of bis(imino)germanone **2**.

Assignment of the Ge=O peak

There are two relevant peaks around 900 cm^{-1} that can be assigned to the Ge=O bond. DFT calculations indicated that Ge=O stretching is at 907 cm^{-1} while there is another notable peak at 861 cm^{-1} that mainly belongs to the C=N moiety of the IPrN groups. We emphasize that the N-Ge π-interactions weakens the degree of Ge=O π-bonding in **2**. However, the phenyl rings in $(Eind)_2Ge=O$ are not perpendicular to the Ge=O bond and as such also have a weakening effect on the Ge=O bond. Therefore, the Ge=O π-bonding in **2** may not be significantly different than that in $(Eind)_2Ge=O$, which can explain the similar IR frequency. This is backed up by the fact that the measured Ge=O bond length is almost identical in **2** (1.6494(10) Å) and in $(Eind)_2Ge=O$ (1.6468(5) Å). Additionally, the detailed orbital analysis shows clear Ge=O π and π* orbitals in **2,** which are barely coupled with the N of the twisted IPrN groups.

Synthesis of compound 3

To the solution of **2** (40 mg, 45 µmol) in C_6D_6 (0.4 mL) in a J. Young PTFE tube, pinacolborane (HBpin) (7 µL, 45 µmol, 1.0 eq.) was added at room temperature. After 45 min, the completion of the reaction was confirmed by ^{1}H NMR. The solvent was removed *in vacuo* to afford **3** as an orange solid (45 mg, 98%).

^{1}H NMR (400 MHz, C_6D_6) δ = 7.26 (t, J = 7.7 Hz, 4H, Ar$\underline{H}$), 7.14 – 7.10 (m, 8H, Ar$\underline{H}$), 5.86 (s, 4H, NC$\underline{H}$), 5.04 (s, 1H, Ge$\underline{H}$), 3.19 – 3.06 (m, 8H, C$\underline{H}$(CH_3)$_2$), 1.24 (d, J = 6.9 Hz, 24H, CH(C$\underline{H}_3$)$_2$), 1.19 (d, J = 6.8 Hz, 12H, CH(C$\underline{H}_3$)$_2$), 1.17 (d, J = 6.8 Hz, 12H, CH(C$\underline{H}_3$)$_2$), 1.18 (s, 6H, BOC(C$\underline{H}_3$)$_2$), 1.11 (s, 6H, BOC(C$\underline{H}_3$)$_2$).

^{13}C NMR (101 MHz, C_6D_6) δ = 154.6 (N$\underline{C}$N), 148.0 (N$\underline{C}$Ar), 147.9 (N$\underline{C}$Ar), 146.5 (N$\underline{C}$Ar), 135.4 (Ar$\underline{C}$), 129.1 (Ar$\underline{C}$), 124.1 (Ar$\underline{C}$), 114.4 (N$\underline{C}$H), 80.2 (BO$\underline{C}$(CH_3)$_2$), 28.8 ($\underline{C}$H(CH_3)$_2$), 25.5 ($\underline{C}H_3$), 24.9 ($\underline{C}H_3$), 24.6 ($\underline{C}H_3$), 24.0 ($\underline{C}H_3$), 23.9 ($\underline{C}H_3$).

^{11}B NMR (128 MHz, C_6D_6) δ = 28.5.

Anal. Calcd. [%] for $C_{60}H_{85}BGeN_6O_3$: C, 70.53; H, 8.39; N, 8.22. Found [%]: C, 70.43; H, 8.50; N, 8.33.

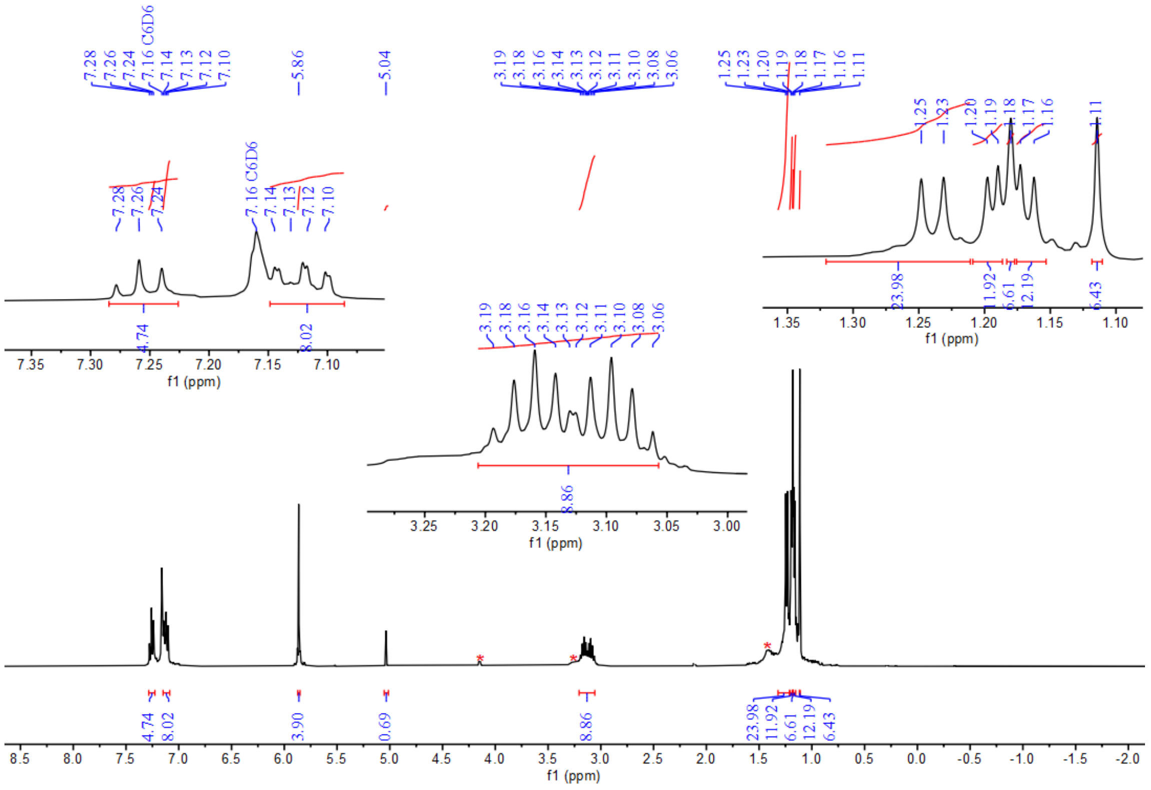

Figure S6. ^{1}H NMR spectrum of compound **3**. The resonances of IPrNH, resulting from minor complex decomposition in solution, are marked with asterisks *.

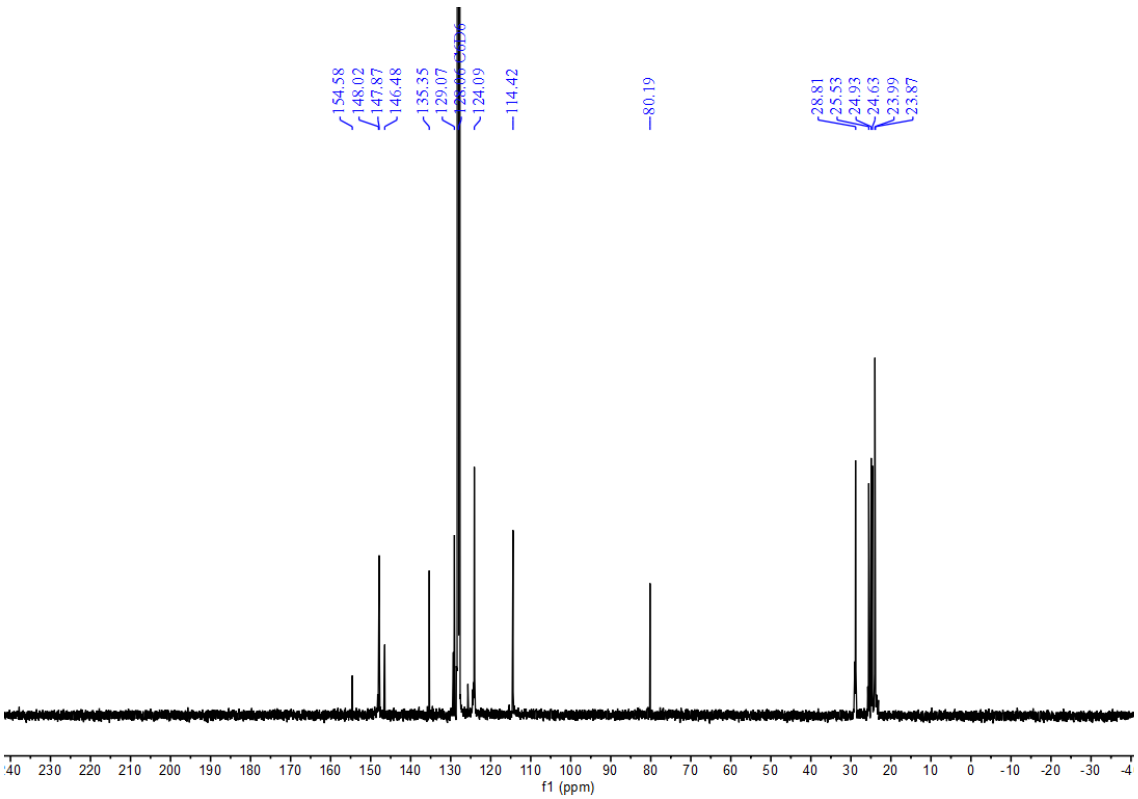

Figure S7. ^{13}C NMR spectrum of compound **3**.

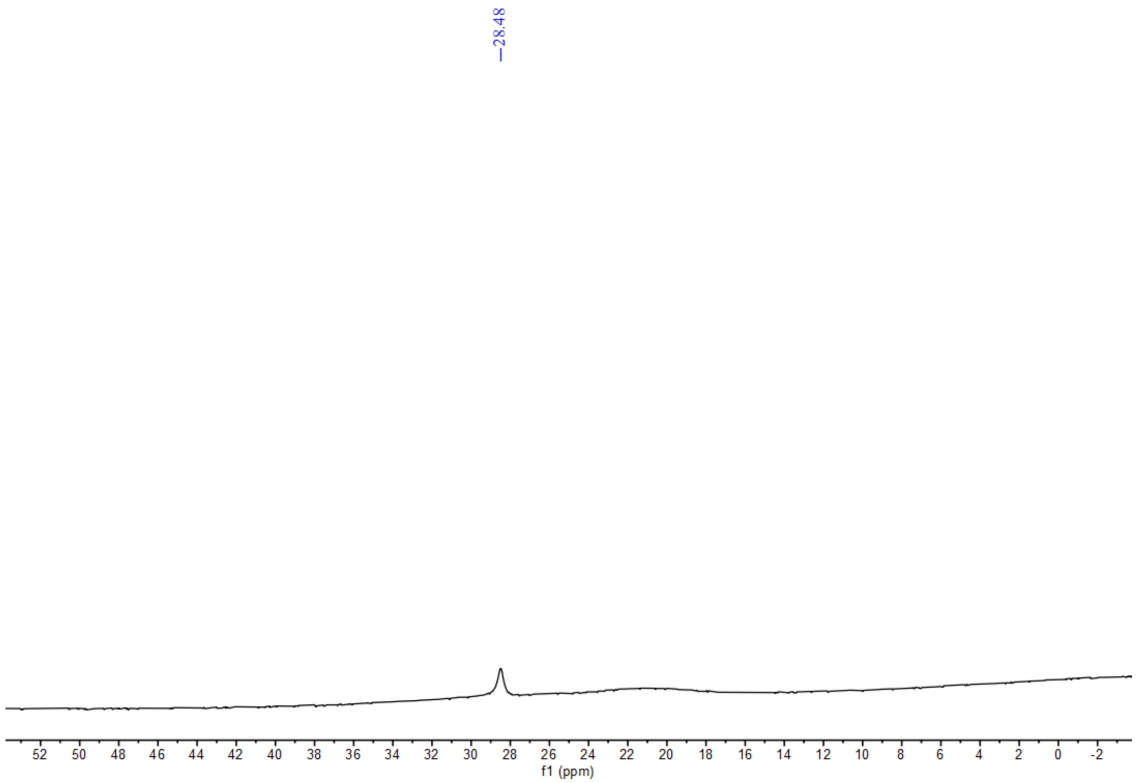

Figure S8. ^{11}B NMR spectrum of compound **3**.

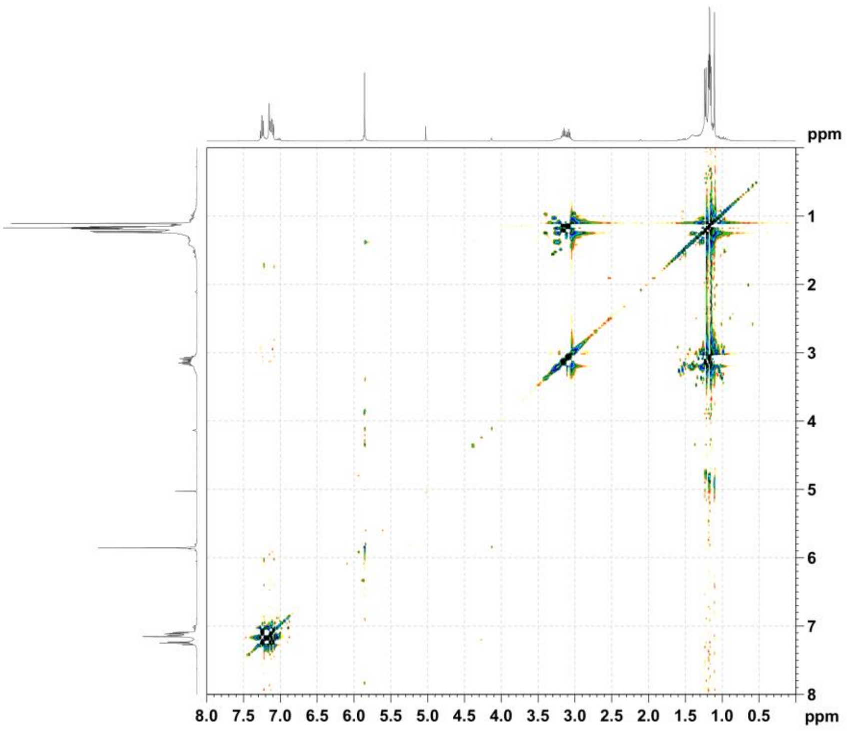

Figure S9. $^{1}H/^{1}H$ COSY NMR spectrum of compound **3**.

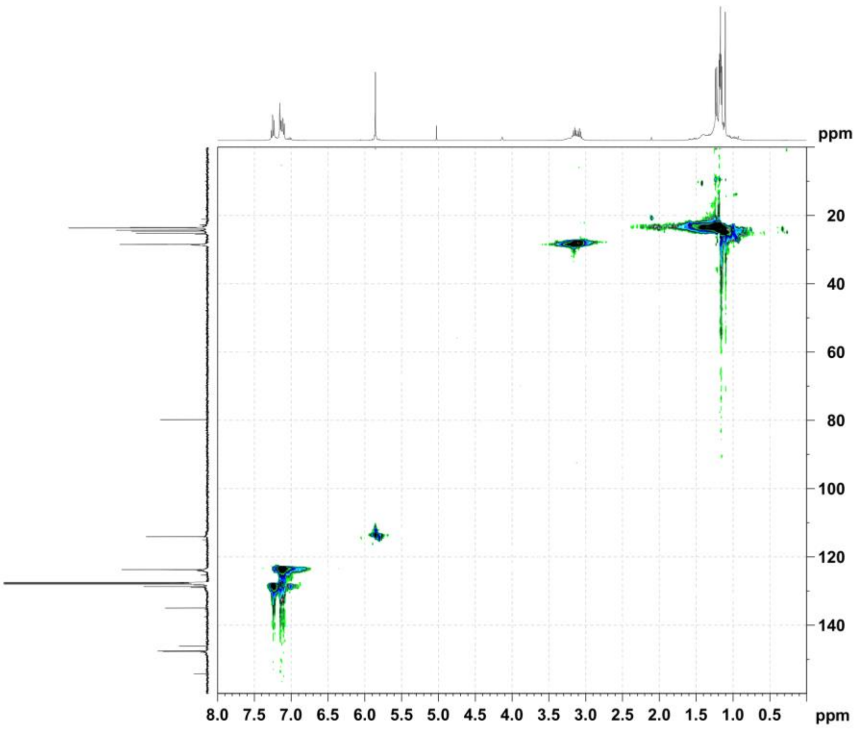

Figure S10. $^{1}H/^{13}C$ HSQC NMR spectrum of compound **3**.

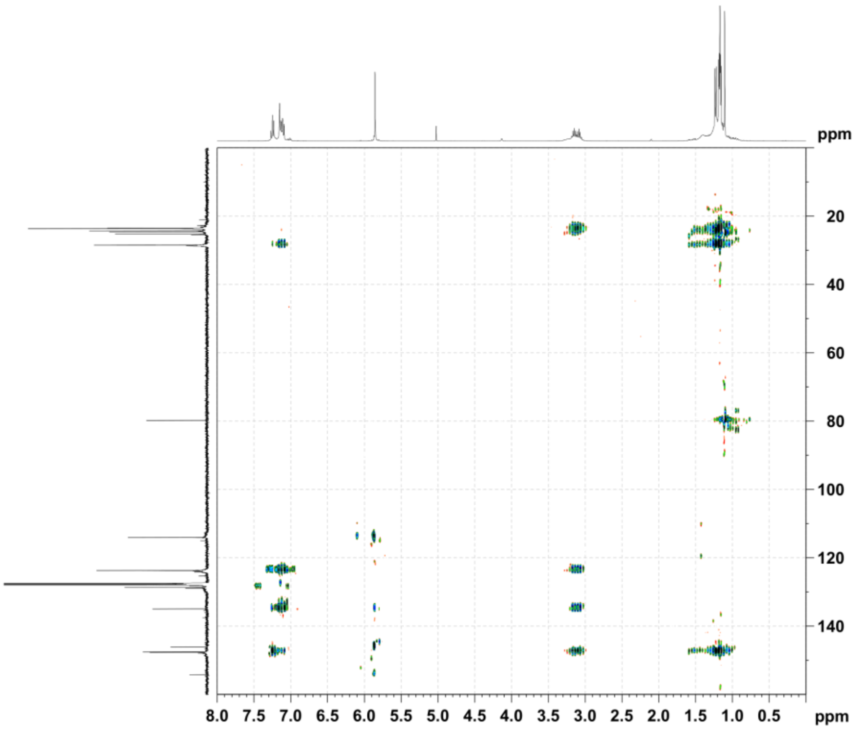

Figure S11. ^{1}H/^{13}C HMBC NMR spectrum of compound **3**.

Synthesis of compound 4

To the solution of **2** (120 mg, 134 μmol) in C_6H_6 (1.2 mL) in a Schlenk tube, bromotrimethylsilane (TMSBr) (18 μL, 134 μmol, 1.0 eq.) was added at room temperature. The reaction mixture was stirred for 20 min. After the reaction, unidentified precipitate was removed by filtration. Then the solvent was removed from the filtrate *in vacuo* to afford **4** as an orange solid (65 mg, 46%).

^{1}H NMR (400 MHz, C_6D_6) δ = 7.24 (t, J = 7.7 Hz, 4H, Ar*H*), 7.13 (d, J = 7.7 Hz, 8H, Ar*H*), 5.87 (s, 4H, NC*H*), 3.25 (septet, J = 6.8 Hz, 4H, C*H*(CH_3)$_2$), 3.09 (septet, J = 6.8 Hz, 4H, C*H*(CH_3)$_2$), 1.36 (d, J = 6.8 Hz, 12H, CH(C*H*$_3$)$_2$), 1.22 (d, J = 6.8 Hz, 12H, CH(C*H*$_3$)$_2$), 1.13 (d, J = 6.8 Hz, 12H, CH(C*H*$_3$)$_2$), 1.11 (d, J = 6.8 Hz, 12H, CH(C*H*$_3$)$_2$), 0.03 (s, 9H, Si(C*H*$_3$)$_3$).

^{13}C NMR (101 MHz, C_6D_6) δ = 148.0 (N*C*N), 147.7 (N*C*Ar), 147.5 (N*C*Ar), 146.3 (N*C*Ar), 135.5 (Ar*C*), 129.3 (Ar*C*), 124.4 (Ar*C*), 123.9 (Ar*C*), 115.8 (N*C*H), 113.8 (N*C*H), 28.9 (*C*H(CH_3)$_2$), 28.7 (*C*H(CH_3)$_2$), 25.4 (CH(*C*H$_3$)$_2$), 24.4 (CH(*C*H$_3$)$_2$), 23.9 (CH(*C*H$_3$)$_2$), 23.7 (CH(*C*H$_3$)$_2$), 23.5 (CH(*C*H$_3$)$_2$), 3.5 (Si(*C*H$_3$)$_3$).

Anal. Calcd. [%] for $C_{57}H_{81}BrGeN_6OSi$: C, 65.39; H, 7.80; N, 8.03. Found [%]: C, 65.30; H, 7.91; N, 8.13.

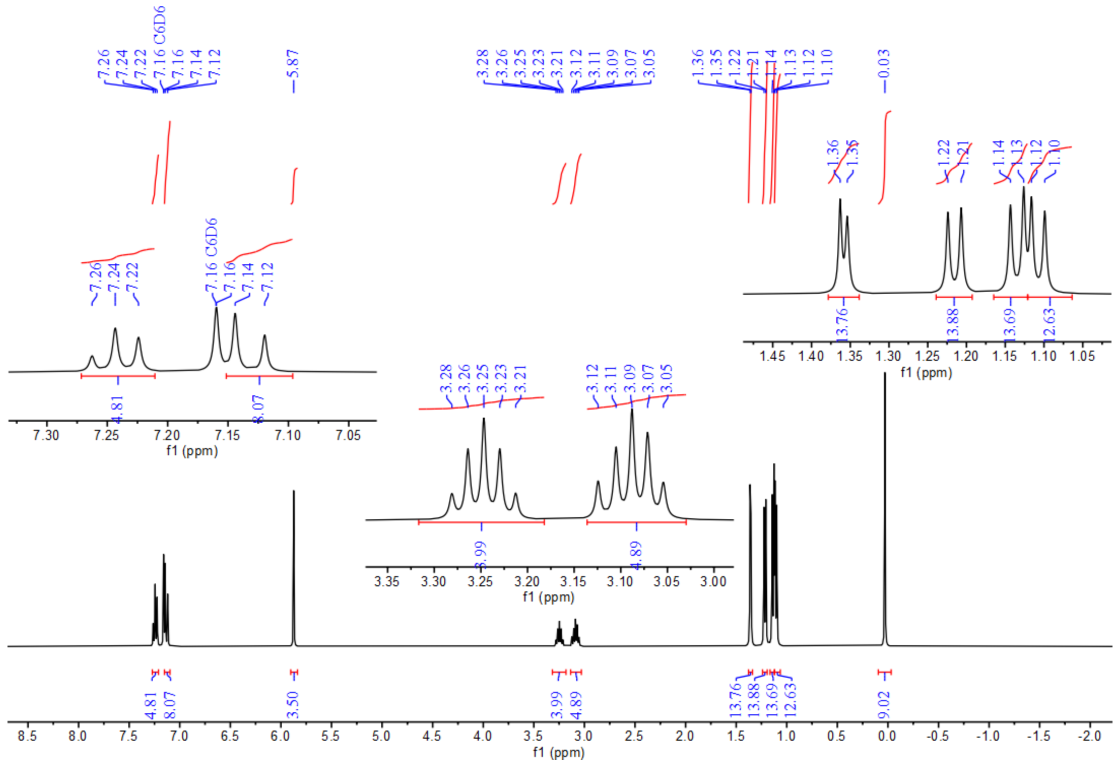

Figure S12. ^{1}H NMR spectrum of compound **4**.

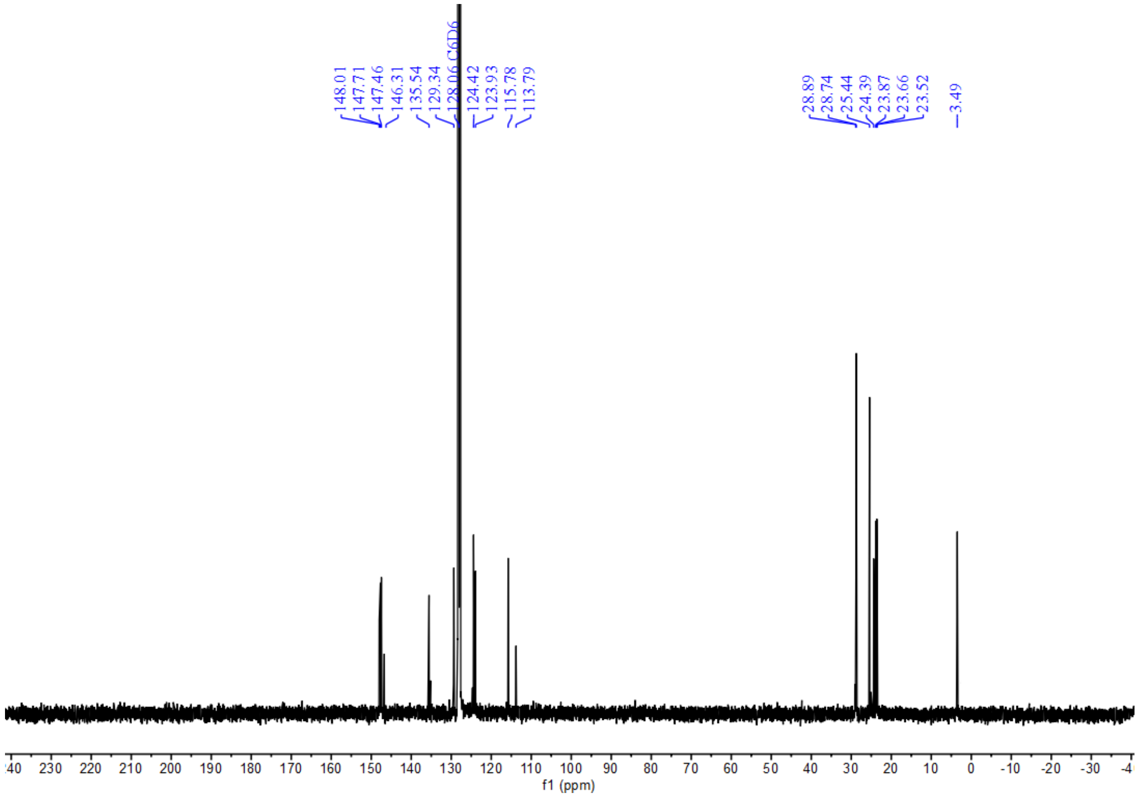

Figure S13. ^{13}C NMR spectrum of compound **4**.

Synthesis of compound 5

To the solution of **2** (200 mg, 224 μmol) in toluene (2.0 mL) in a Schlenk tube, phenylsilane ($PhSiH_3$) (28 μL, 224 μmol, 1.0 eq.) was added at room temperature. The reaction mixture was stirred for 30 min. The solvent was removed *in vacuo* to afford **5** as an orange solid (180 mg, 80%).

^{1}H NMR (400 MHz, C_6D_6) δ = 7.63 – 7.61 (m, 2H, Ar<u>H</u>), 7.28 – 7.22 (m, 7H, Ar<u>H</u>), 7.13 – 7.06 (m, 8H, Ar<u>H</u>), 5.88 (s, 4H, NC<u>H</u>), 5.09 (s, 2H, Si$\underline{H}_2$), 4.78 (s, 1H, Ge<u>H</u>), 3.18 – 3.04 (m, 8H, C<u>H</u>(CH_3)$_2$), 1.15 – 1.11 (m, 48H, CH(C$\underline{H}_3$)$_2$).

^{13}C NMR (101 MHz, C_6D_6) δ = 154.6 (N<u>C</u>N), 148.2 (N<u>C</u>Ar), 147.9 (N<u>C</u>Ar), 147.1 (N<u>C</u>Ar), 138.7 (SiH_2<u>C</u>), 135.0 (SiH_2C<u>C</u>H), 134.8 (Ar<u>C</u>), 129.2 (Ar<u>C</u>), 124.0 (Ar<u>C</u>), 114.4 (N<u>C</u>H), 28.9 (<u>C</u>H(CH_3)$_2$), 28.7 (<u>C</u>H(CH_3)$_2$), 24.9 (CH(<u>C</u>H_3)$_2$), 23.8 (CH(<u>C</u>H_3)$_2$), 23.4 (CH(<u>C</u>H_3)$_2$).

Anal. Calcd. [%] for $C_{60}H_{80}GeN_6OSi$: C, 71.92; H, 8.05; N, 8.39. Found [%]: C, 71.87; H, 8.15; N, 8.44.

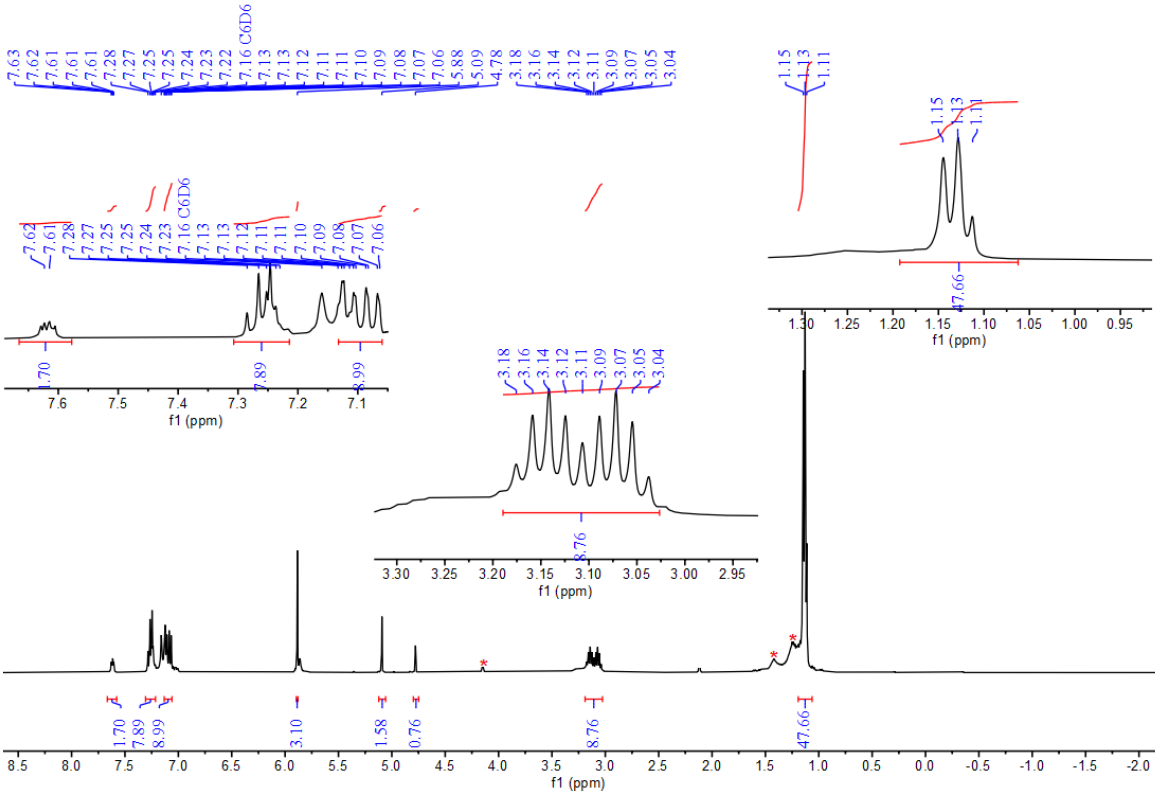

Figure S14. ^{1}H NMR spectrum of compound **5**. The resonances of IPrNH, resulting from minor complex decomposition in solution, are marked with asterisks *.

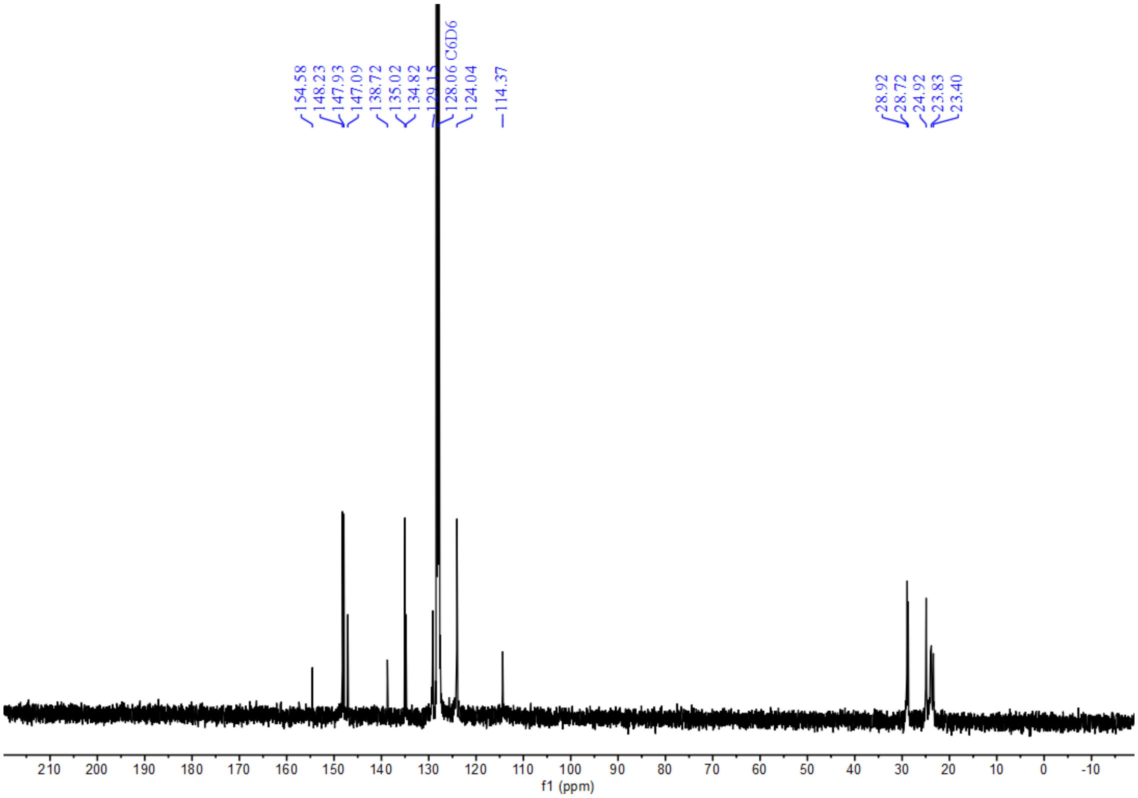

Figure S15. ^{13}C NMR spectrum of compound **5**.

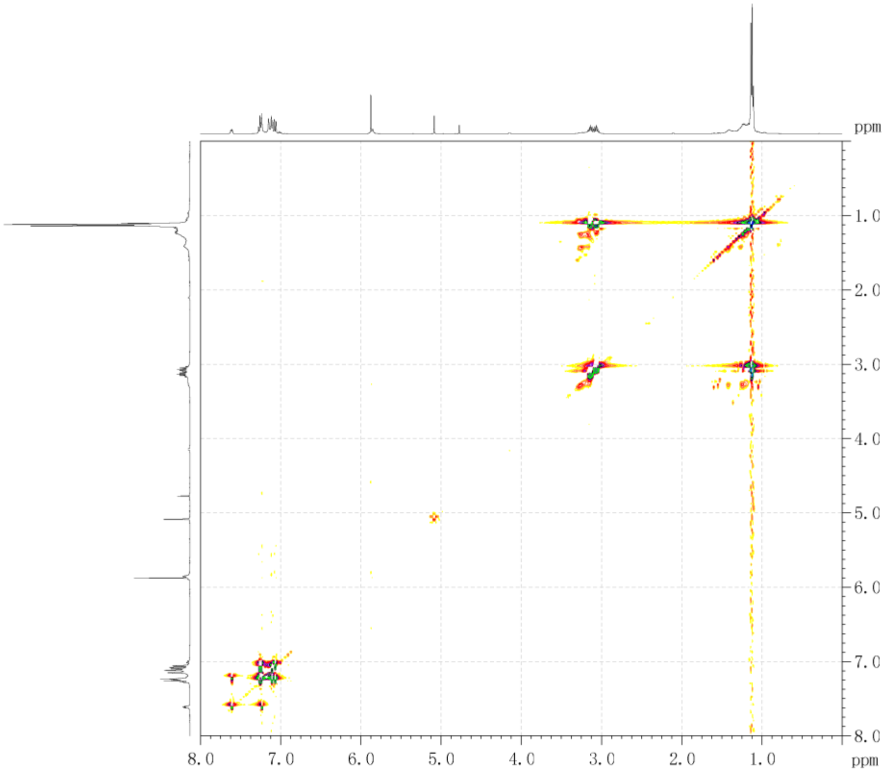

Figure S16. ^{1}H/^{1}H COSY NMR spectrum of compound **5**.

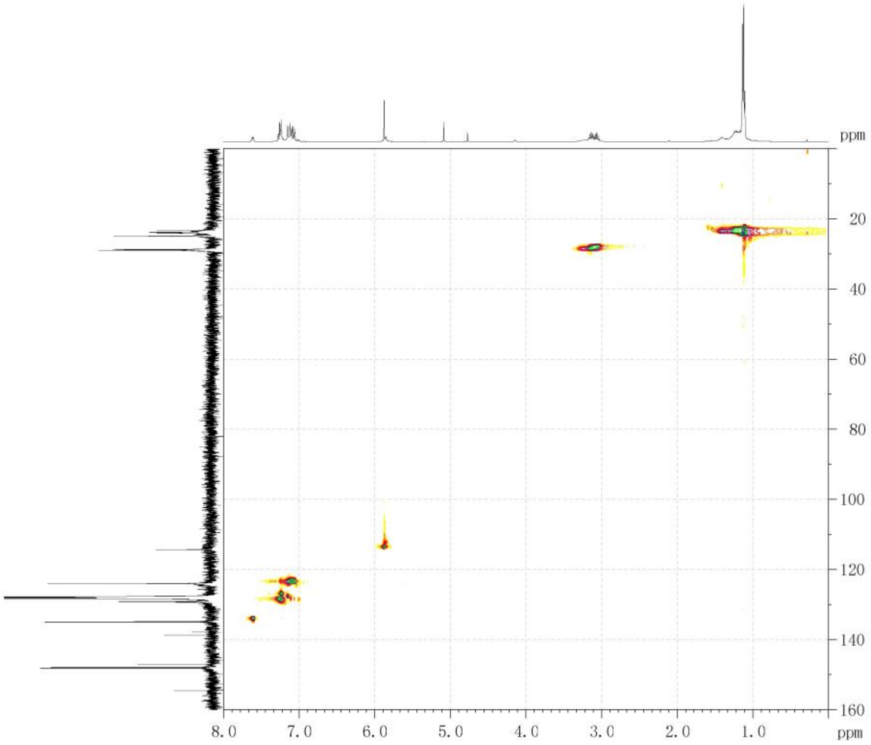

Figure S17. $^{1}H/^{13}C$ HSQC NMR spectrum of compound **5**.

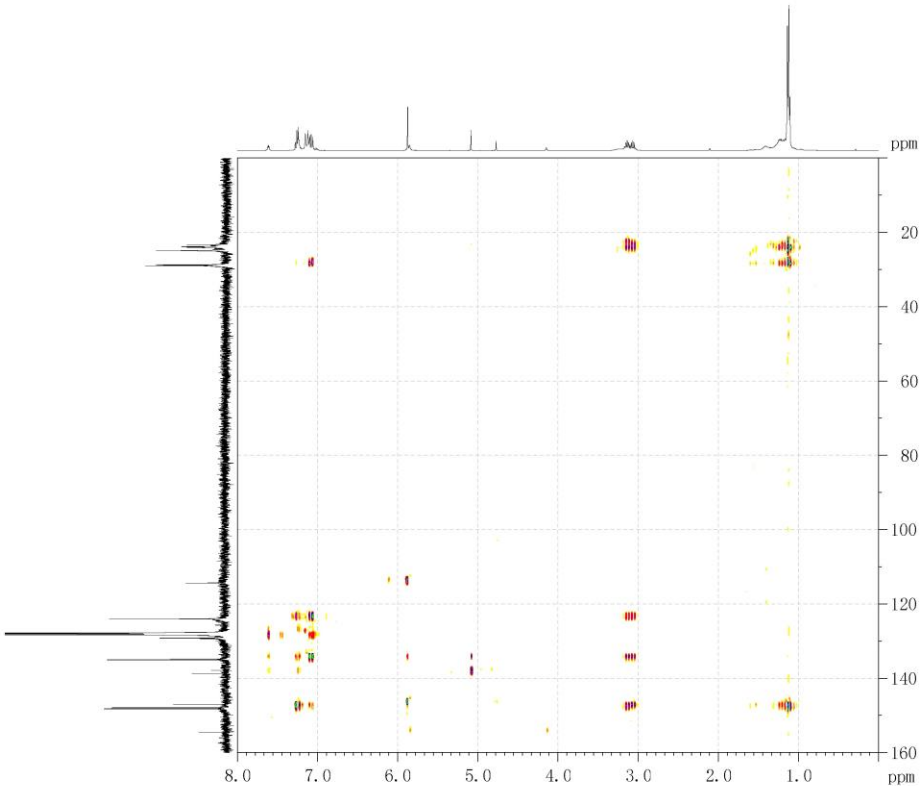

Figure S18. $^{1}H/^{13}C$ HMBC NMR spectrum of compound **5**.

Synthesis of compound 6

To the solution of **2** (200 mg, 224 μmol) in toluene (2.0 mL) in a Schlenk tube, benzaldehyde (PhCHO) (23 μL, 224 μmol, 1.0 eq.) was added at room temperature. The reaction mixture was stirred for 30 min. The solvent was removed *in vacuo* to afford **6** as an orange solid (155 mg, 70%).

^{1}H NMR (400 MHz, C_6D_6) δ = 7.47 (d, J = 6.9 Hz, 2H, Ar*H*), 7.28 – 7.22 (m, 7H, Ar*H*), 7.13 – 7.09 (m, 8H, Ar*H*), 6.01 (s, 2H, NC*H*), 5.96 (s, 2H, NC*H*), 5.10 (s, 1H, OC*H*), 3.07 (septet, J = 6.8 Hz, 8H, C*H*(CH_3)$_2$), 1.19 (d, J = 6.8 Hz, 12H, CH(C*H*$_3$)$_2$), 1.14 (d, J = 6.8 Hz, 12H, CH(C*H*$_3$)$_2$), 1.11 (d, J = 6.8 Hz, 12H, CH(C*H*$_3$)$_2$), 0.96 (d, J = 6.8 Hz, 12H, CH(C*H*$_3$)$_2$).

^{13}C NMR (101 MHz, C_6D_6) δ = 154.6 (N*C*N), 148.3 (N*C*Ar), 148.0 (N*C*Ar), 147.8 (N*C*Ar), 147.7 (N*C*Ar), 146.2 (N*C*Ar), 134.5 (Ar*C*), 134.0 (Ar*C*), 129.2 (Ar*C*), 127.2 (Ar*C*), 124.1 (Ar*C*), 114.9 (N*C*H), 99.7 (O*C*HO), 29.0 (*C*H(CH_3)$_2$), 25.4 (CH(*C*H$_3$)$_2$), 25.1 (CH(*C*H$_3$)$_2$), 24.0 (CH(*C*H$_3$)$_2$), 23.2 (CH(*C*H$_3$)$_2$).

Anal. Calcd. [%] for $C_{61}H_{78}GeN_6O_2$: C, 73.27; H, 7.86; N, 8.40. Found [%]: C, 72.55; H, 8.00; N, 8.40.

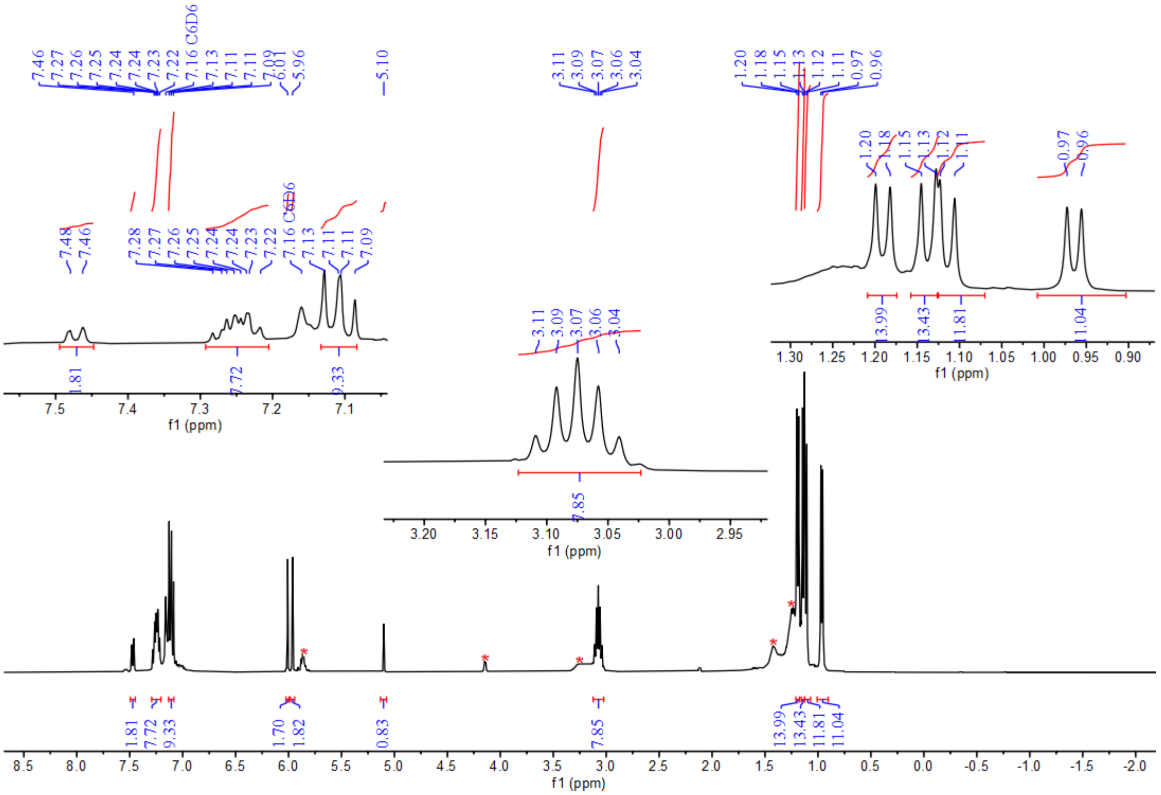

Figure S19. ^{1}H NMR spectrum of compound **6**. The resonances of IPrNH, resulting from minor complex decomposition in solution, are marked with asterisks *.

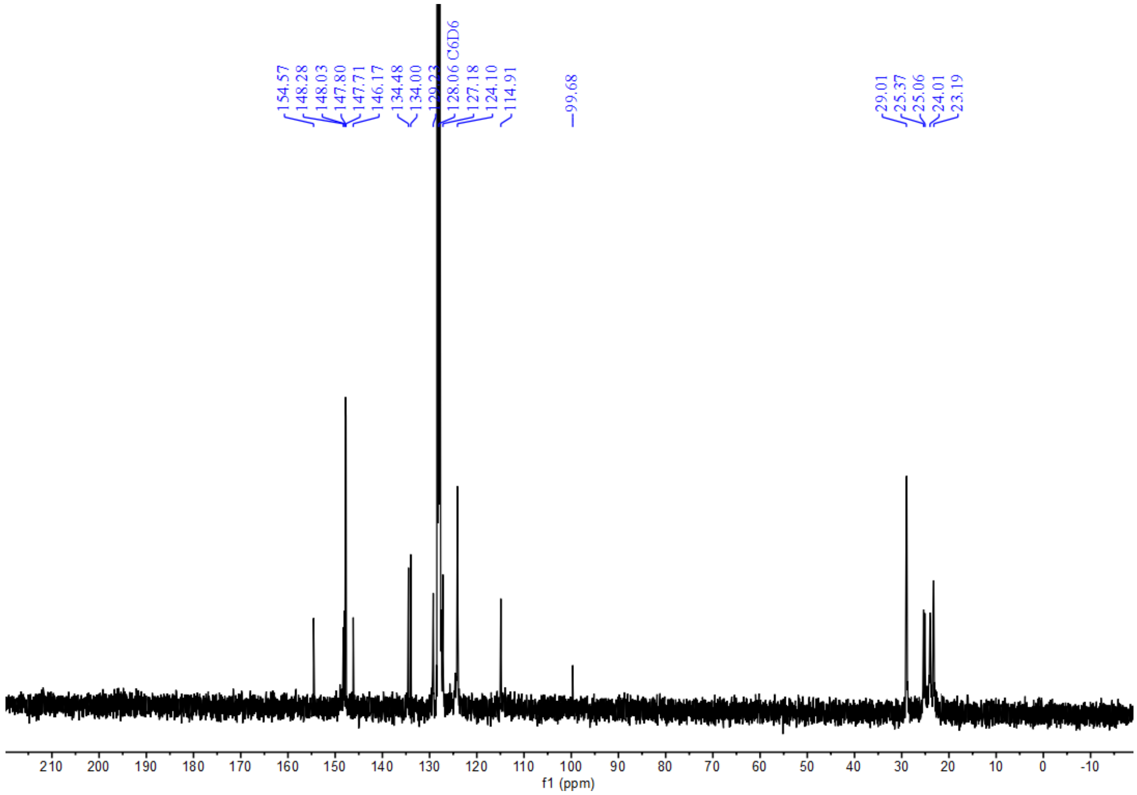

Figure S20. ^{13}C NMR spectrum of compound **6**.

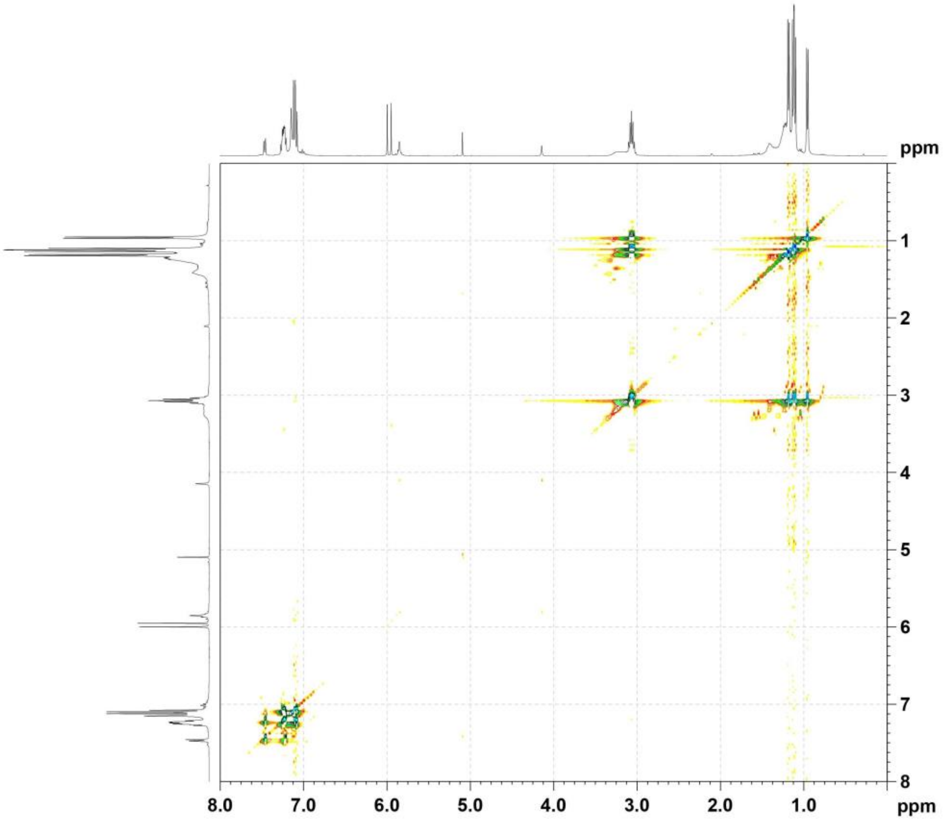

Figure S21. $^{1}H/^{1}H$ COSY NMR spectrum of compound **6**.

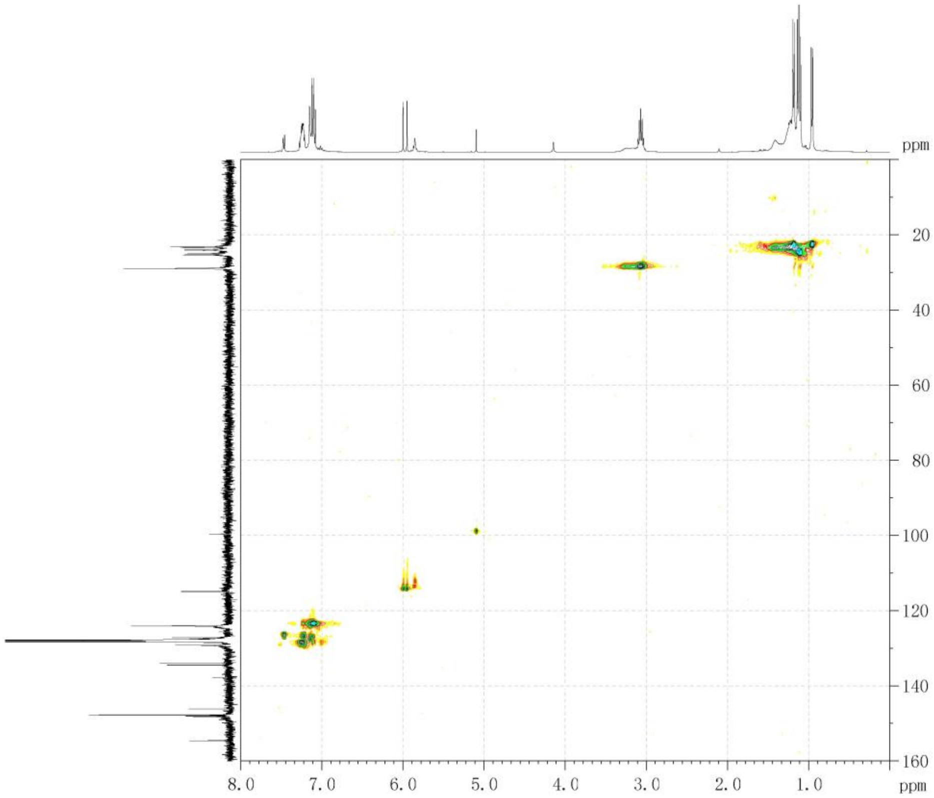

Figure S22. $^{1}H/^{13}C$ HSQC NMR spectrum of compound **6**.

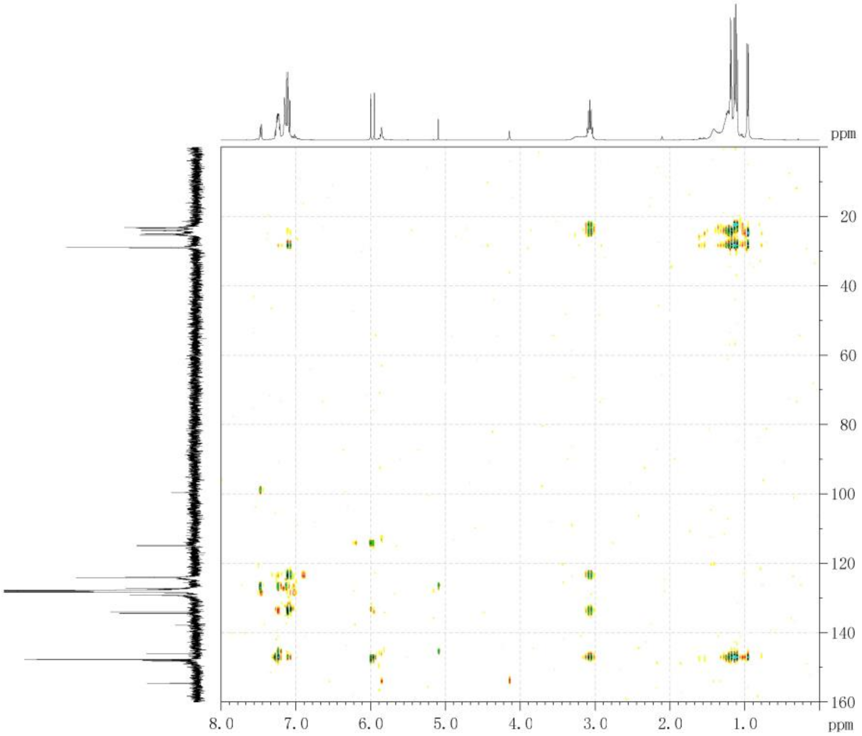

Figure S23. $^{1}H/^{13}C$ HMBC NMR spectrum of compound **6**.

Synthesis of compound 7

To the solution of **2** (200 mg, 224 μmol) in toluene (2.0 mL) in a Schlenk tube, phenylacetylene (PhCCH) (25 μL, 224 μmol, 1.0 eq.) was added at room temperature. The reaction mixture was stirred for 30 min. The solvent was removed *in vacuo* to afford **7** as an orange solid (180 mg, 81%).

^{1}H NMR (400 MHz, C_6D_6) δ = 7.42 (d, J = 7.0 Hz, 2H, Ar$\underline{H}$), 7.29 – 7.18 (m, 7H, Ar$\underline{H}$), 7.13 – 7.10 (m, 8H, Ar$\underline{H}$), 5.86 (s, 4H, NC$\underline{H}$), 3.19 – 3.10 (m, 8H, C$\underline{H}$(CH$_3$)$_2$), 1.26 (d, J = 6.8 Hz, 12H, CH(C$\underline{H}_3$)$_2$), 1.23 (d, J = 6.8 Hz, 12H, CH(C$\underline{H}_3$)$_2$), 1.17 (d, J = 6.8 Hz, 12H, CH(C$\underline{H}_3$)$_2$), 1.15 (d, J = 6.8 Hz, 12H, CH(C$\underline{H}_3$)$_2$), -0.77 (s, 1H, GeO$\underline{H}$).

^{13}C NMR (101 MHz, C_6D_6) δ = 154.6 (N$\underline{C}$N), 148.3 (N$\underline{C}$Ar), 148.2 (N$\underline{C}$Ar), 145.6 (N$\underline{C}$Ar), 135.1 (Ar$\underline{C}$), 132.4 (Ar$\underline{C}$), 129.3 (Ar$\underline{C}$), 124.0 (Ar$\underline{C}$), 114.3 (N$\underline{C}$H), 98.0 (GeC$\underline{C}$Ph), 96.6 (Ge$\underline{C}$CPh), 29.0 ($\underline{C}$H(CH$_3$)$_2$), 24.6 (CH($\underline{C}$H$_3$)$_2$), 23.9 (CH($\underline{C}$H$_3$)$_2$).

Anal. Calcd. [%] for $C_{62}H_{78}GeN_6O$: C, 74.77; H, 7.89; N, 8.44. Found [%]: C, 74.97; H, 7.85; N, 7.94.

LIFDI-MS: calculated for $C_{62}H_{78}GeN_6O$: 996.54489. Found: 996.54934.

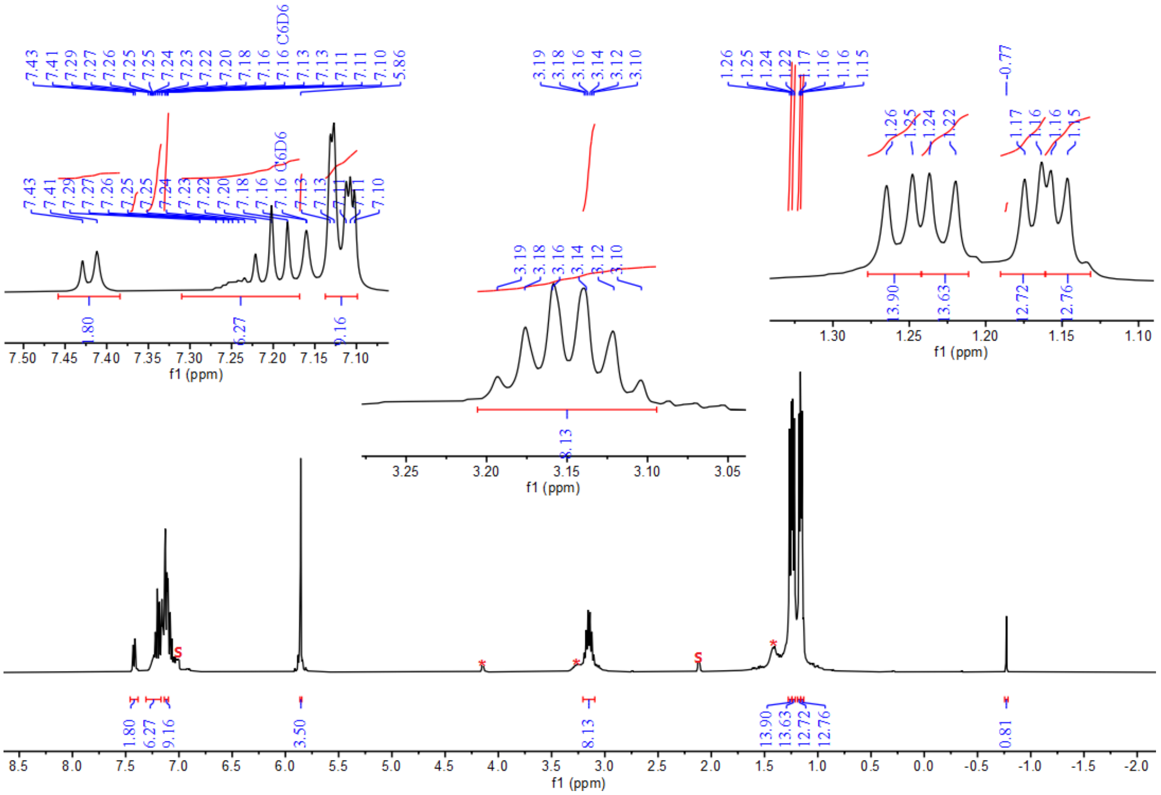

Figure S24. ^{1}H NMR spectrum of compound **7**. The respective resonances of IPrNH, resulting from minor complex decomposition in solution, are marked with asterisks * and residual toluene with an S.

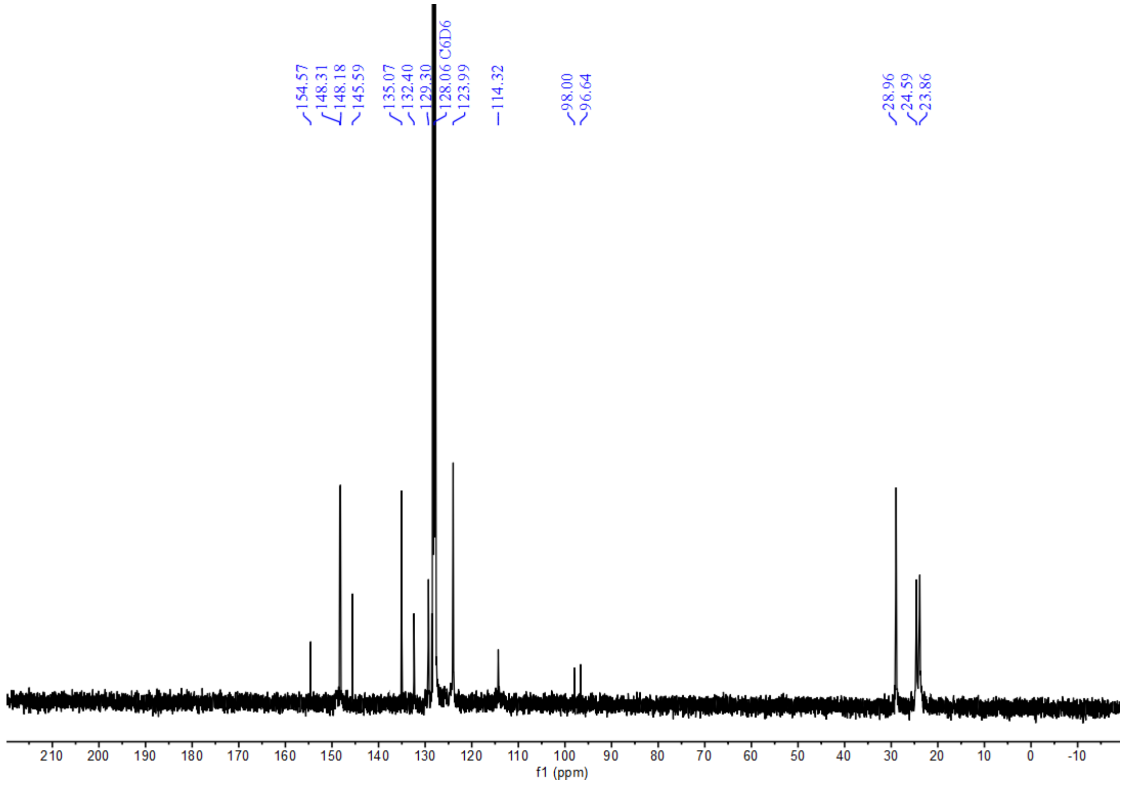

Figure S25. ^{13}C NMR spectrum of compound **7**.

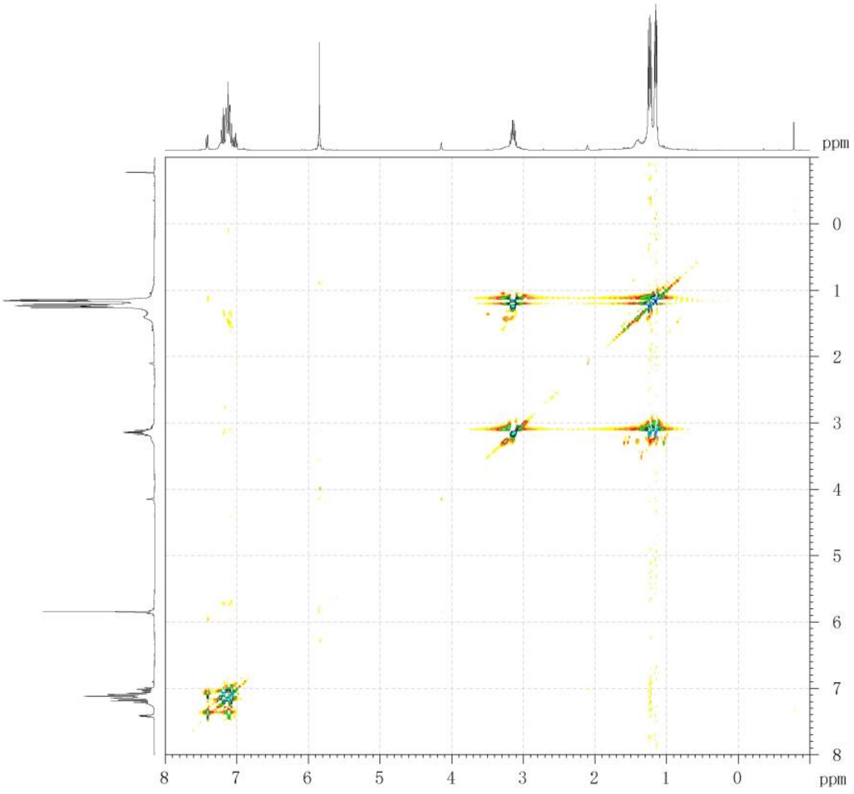

Figure S26. ^{1}H/^{1}H COSY NMR spectrum of compound **7**.

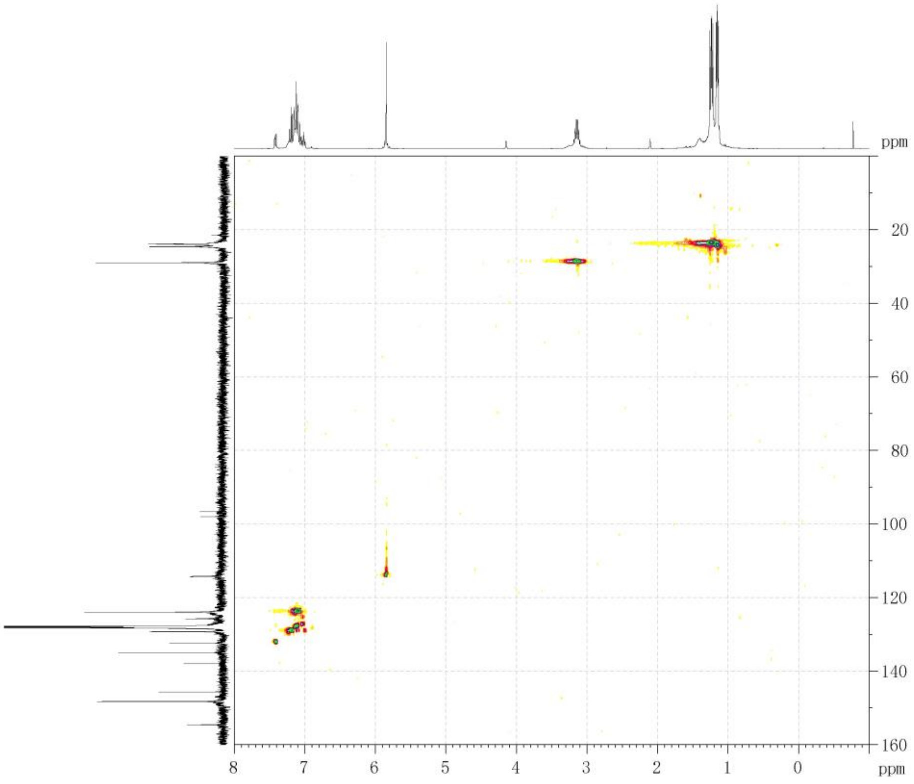

Figure S27. $^{1}H/^{13}C$ HSQC NMR spectrum of compound **7**.

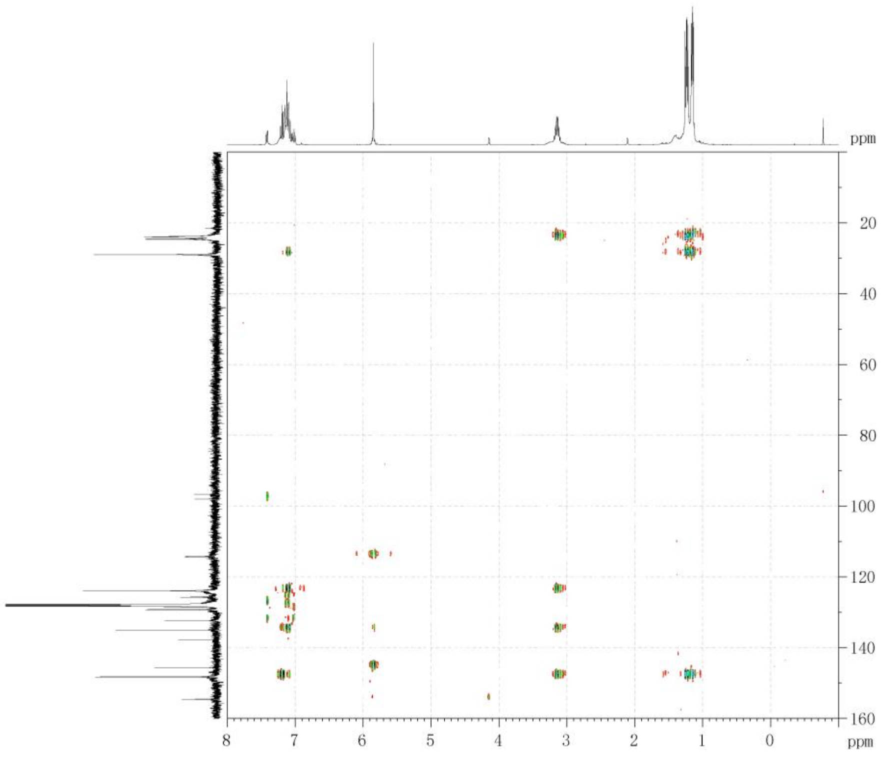

Figure S28. $^{1}H/^{13}C$ HMBC NMR spectrum of compound **7**.

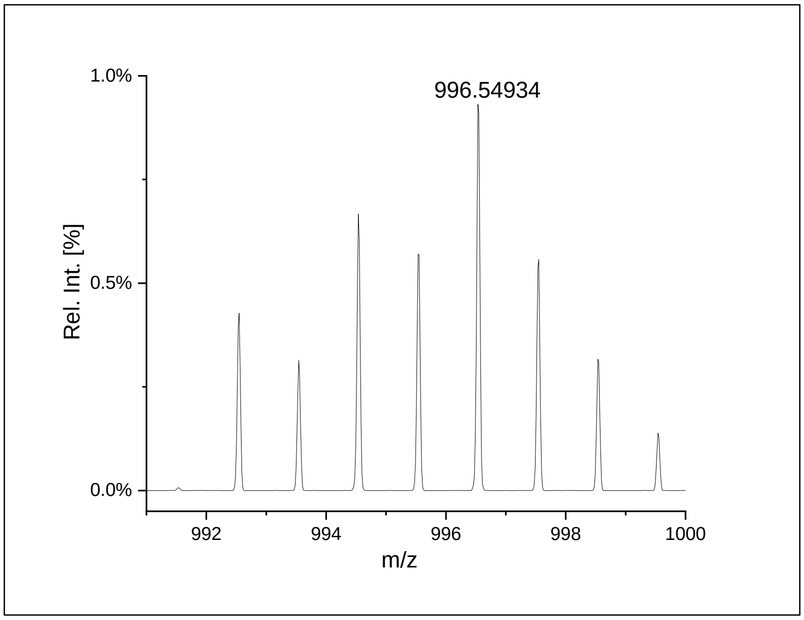

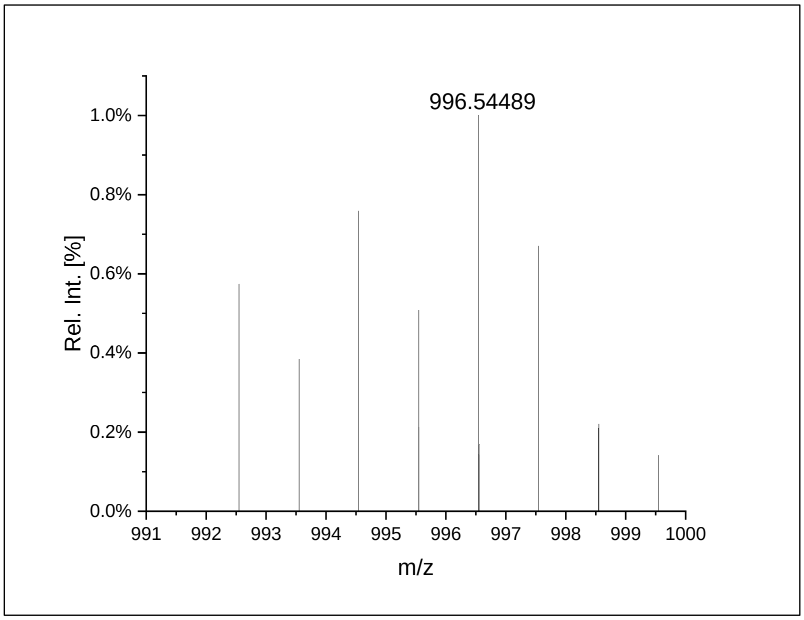

Figure S29. LIFDI-MS spectrum: expanded region of the compound signal showing the isotopic pattern of compound **7**. Measured (top) and calculated (bottom).

Reaction of 2 and 2,6-dimethylphenyl isocyanide (CNXyl)

To the mixture of **2** (40 mg, 45 μmol) and 2,6-dimethylphenyl isocyanide (CNXyl) (3 mg, 22 μmol, 0.5 eq.) in a J. Young PTFE tube, C_6D_6 (0.4 mL) was added at room temperature. The reaction mixture was heated at 80 ^{o}C. After 2.0 h, the completion of the reaction was confirmed by ^{1}H NMR spectroscopy.

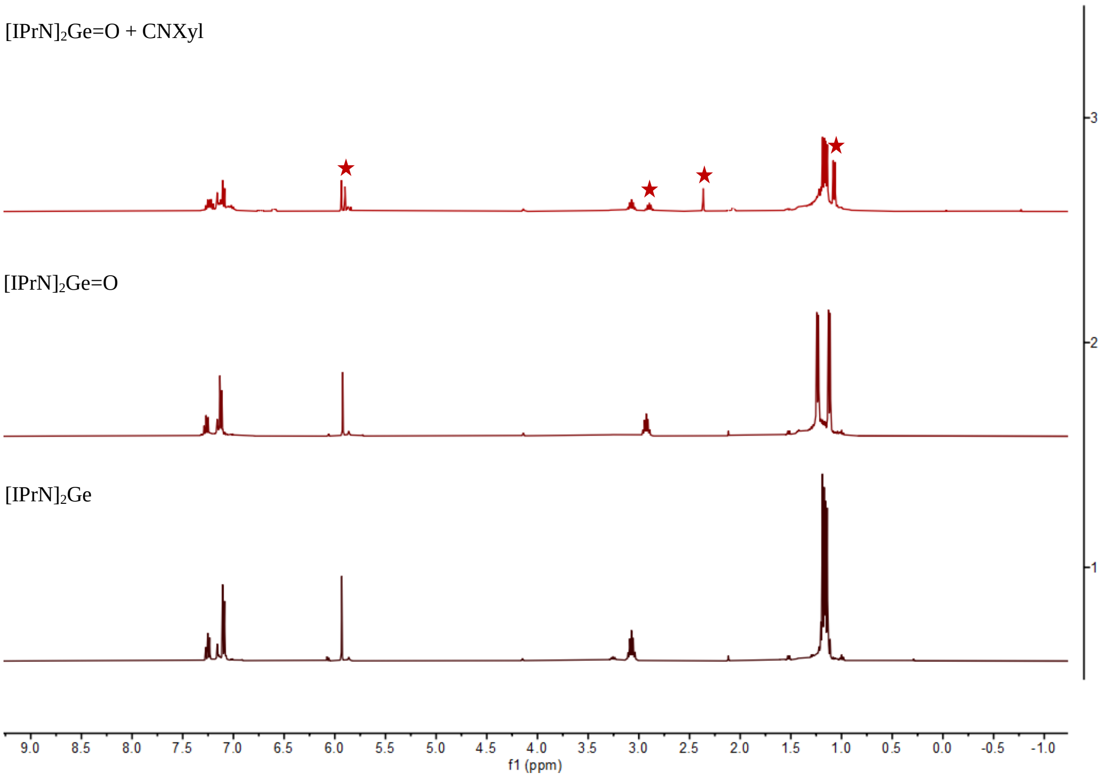

Figure S30. ^{1}H NMR spectrum of germylene **1**, germanone **2**, as well as the product mixture of germylene **1** and [2+2] cycloaddition product **8** (★).

Synthesis of compound 8

To the solution of **2** (40 mg, 45 µmol) in C_6D_6 (0.4 mL) in a J. Young PTFE tube, 2,6-dimethylphenyl isocyanate (7 µL, 45 µmol, 1.0 eq.) was added at room temperature. After 30 min, the completion of the reaction was confirmed by ^{1}H NMR. The solvent was removed *in vacuo* to afford **8** as an orange solid (44 mg, 90%).

^{1}H NMR (400 MHz, C_6D_6) δ = 7.22 (t, J = 7.7 Hz, 4H, Ar$\underline{H}$), 7.14 (d, J = 7.7 Hz, 8H, Ar$\underline{H}$), 7.02 – 6.97 (m, 3H, Ar$\underline{H}$), 5.90 (s, 4H, NC$\underline{H}$), 2.90 (septet, J = 6.8 Hz, 8H, C$\underline{H}$(CH$_3$)$_2$), 2.37 (s, 6H, ArC$\underline{H}_3$), 1.22 – 1.13 (m, 24H, CH(C$\underline{H}_3$)$_2$), 1.07 (d, J = 6.8 Hz, 24H, CH(C$\underline{H}_3$)$_2$).

^{13}C NMR (101 MHz, C_6D_6) δ = 154.6 (N$\underline{C}$N), 153.0 (O$\underline{C}$N), 148.6 (N$\underline{C}$Ar), 147.2 (N$\underline{C}$Ar), 133.3 (Ar$\underline{C}$), 130.9 (Ar$\underline{C}$), 129.8 (Ar$\underline{C}$), 124.5 (Ar$\underline{C}$), 115.1 (N$\underline{C}$H), 29.0 ($\underline{C}$H(CH$_3$)$_2$), 25.2 (CH($\underline{C}$H$_3$)$_2$), 24.0 (CH($\underline{C}$H$_3$)$_2$), 23.4 (CH($\underline{C}$H$_3$)$_2$), 23.2 (CH($\underline{C}$H$_3$)$_2$), 19.9 (Ar$\underline{C}$H$_3$).

Anal. Calcd. [%] for $C_{63}H_{81}GeN_7O_2$: C, 72.69; H, 7.84; N, 9.42. Found [%]: C, 72.87; H, 7.85; N, 9.64.

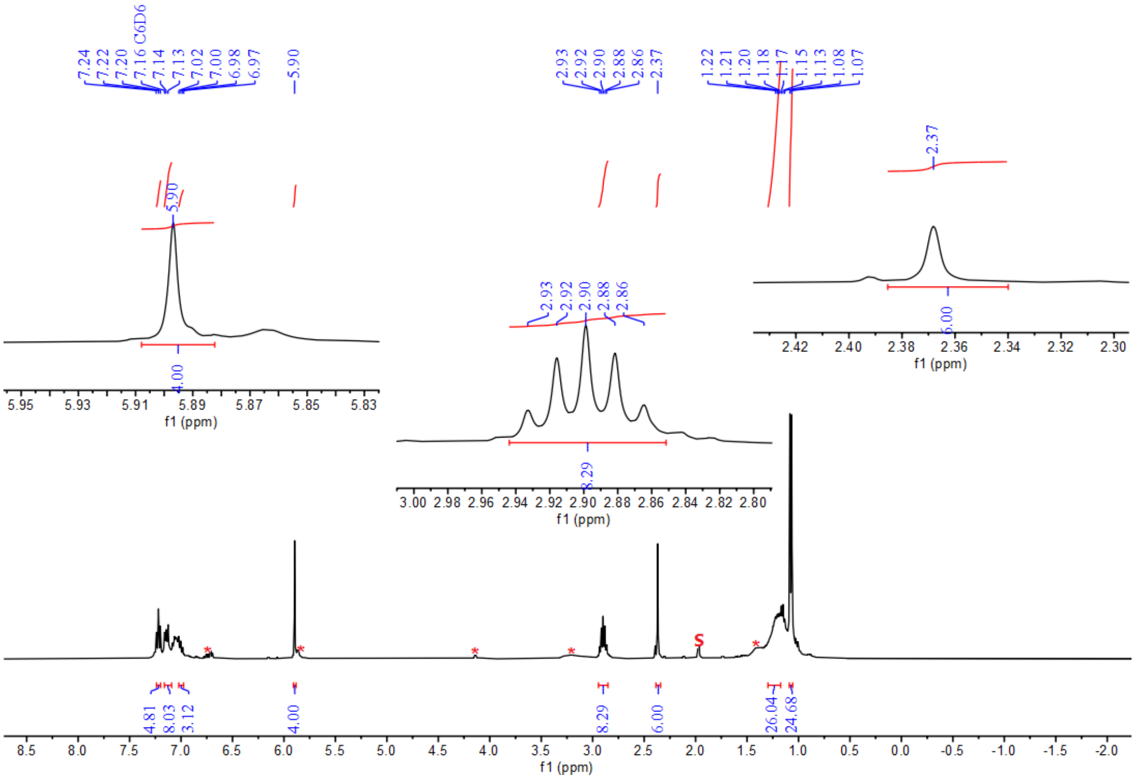

Figure S31. ^{1}H NMR spectrum of compound **8**. The respective resonances of IPrNH, resulting from minor complex decomposition in solution, are marked with asterisks * and residual 2,6-dimethylphenyl isocyanate with an S.

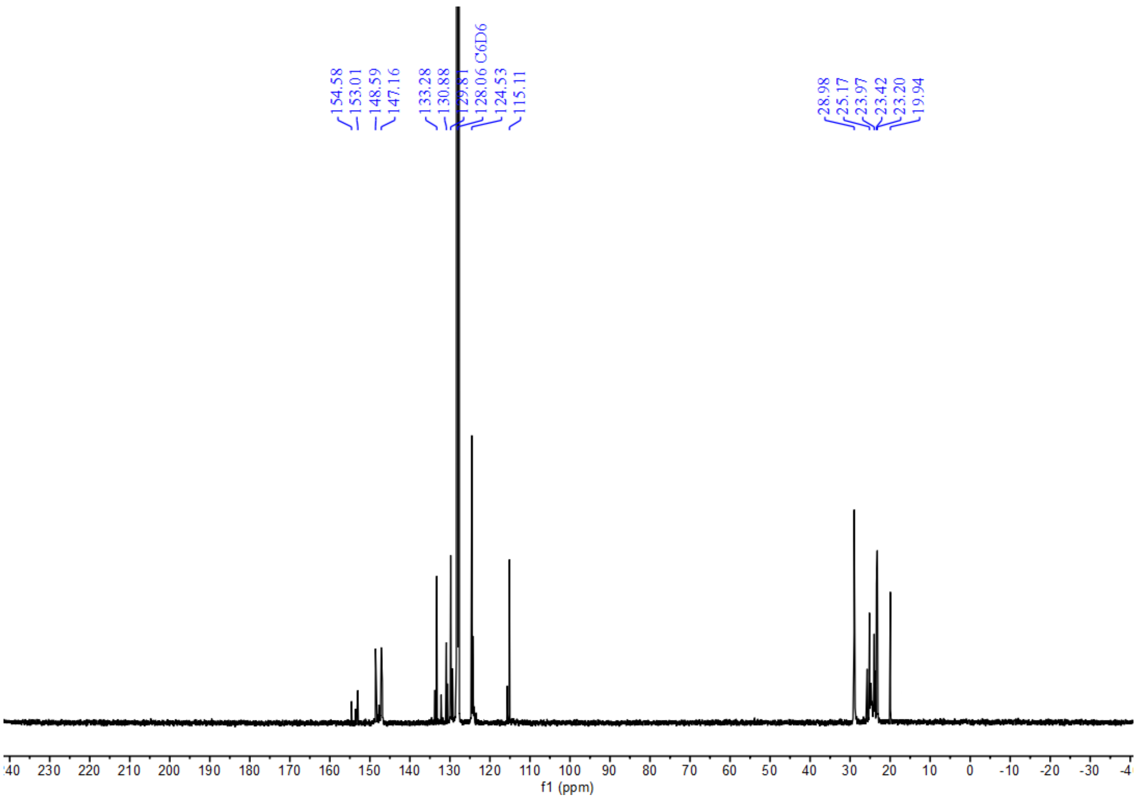

Figure S32. ^{13}C NMR spectrum of compound **8**.

Reaction of 2 and methanol (MeOH)

To the solution of **2** (40 mg, 45 μmol) in C_6D_6 (0.4 mL) in a J. Young PTFE tube, methanol (MeOH) (2 μL, 45 μmol, 1.0 eq.) was added at room temperature. The color rapidly changed from orange to yellow. The product was confirmed by ^{1}H NMR spectrum as the free ligand IPrNH.

2. X-ray Crystallographic Data

General Information

The X-ray intensity data of **2** was collected on an X-ray single crystal diffractometer equipped with a CMOS detector (Bruker Photon-100), an IMS microsource with MoKα radiation (λ = 0.71073 Å) and a Helios mirror optic by using the APEX III software package.[S3] The measurement was performed on single crystals coated with the perfluorinated ether Fomblin® Y. The crystal was fixed on the top of a microsampler, transferred to the diffractometer and frozen under a stream of cold nitrogen. A matrix scan was used to determine the initial lattice parameters. Reflections were merged and corrected for Lorenz and polarization effects, scan speed, and background using SAINT.[S4] Absorption corrections, including odd and even ordered spherical harmonics were performed using SADABS.[S4] Space group assignments were based upon systematic absences, E statistics, and successful refinement of the structures. Structures were solved by direct methods with the aid of successive difference Fourier maps, and were refined against all data using the APEX III software in conjunction with SHELXL-2014[S5] and SHELXLE.[S6] All H atoms were placed in calculated positions and refined using a riding model, with methylene and aromatic C–H distances of 0.99 and 0.95 Å, respectively, and Uiso(H) = $1.2 \cdot U_{eq}$(C). Full-matrix least-squares refinements were carried out by minimizing $\Sigma w(Fo^2–Fc^2)^2$ with SHELXL-97[S7] weighting scheme. Neutral atom scattering factors for all atoms and anomalous dispersion corrections for the non-hydrogen atoms were taken from International Tables for Crystallography.[S8] The image of the crystal structure was generated by Mercury.[S9] The CCDC number 2053427 (**2**) contains the supplementary crystallographic data for the structure. The data can be obtained free of charge from the Cambridge Crystallographic Data Centre *via* https://www.ccdc.cam.ac.uk/structures/.

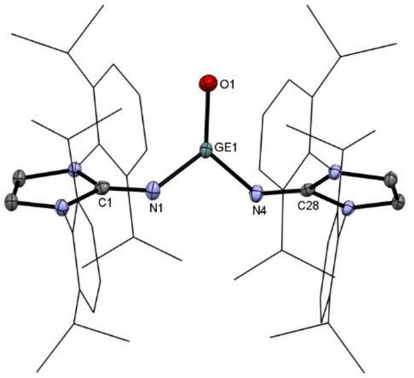

Figure S33. Molecular structure of **2**. Thermal ellipsoids are shown at 50% probability level. Hydrogen atoms are omitted for clarity. Selected bond lengths [Å] and angles [°]: Ge1–O1 1.6494(10), Ge1–N1 1.7819(12), Ge1–N4 1.7825(12), N1–C1 1.2872(18), N4–C28 1.2914(18), O1–Ge1–N1 125.94(6), O1–Ge1–N4 125.41(6), N1–Ge1–N4 108.65(6), Ge1–N1–C1 127.81(10), Ge1–N4–C28 125.02(10).

Table S1. Crystal data and structure refinement for compound **2**.

Compound #	2
Chemical formula	$C_{54} H_{72} Ge N_6 O$
Formula weight	893.79 g/mol
Temperature	100 K
Wavelength	0.71073 Å
Crystal size	0.356 x 0.230 x 0.180 mm
Crystal habit	clear light-yellow block
Crystal system	monoclinic
Space group	P 21/c
Unit cell dimensions	a = 21.8129(7) Å; α = 90°
	b = 11.8549(4) Å; β = 104.710(1)°
	c = 19.9092(7) Å; γ = 90°
Volume	4979.6(3) $Å^3$
Z	4
Density (calculated)	1.192 g/cm^3
Radiation source	IMS microsource
Theta range for data collection	1.97 to 25.35°
Index ranges	-26<=h<=26, -14<=k<=14, -23<=l<=23
Reflections collected	161254
Independent reflections	9095
Completeness	0.999
Absorption correction	Multi-Scan
Max. and min. transmission	0.7099 and 0.7452
Refinement method	Full-matrix least-squares on F^2
Function minimized	Σ w(F_o^2 - F_c^2)^2
Data / restraints / parameters	9095 / 0 / 575
Goodness-of-fit on F^2	1.035
Final R indices [I>2sigma(I)]	R1 = 0.0248, wR2 = 0.0613
R indices (all data)	R1 = 0.0288, wR2 = 0.0644
Largest diff. peak and hole	0.264 and -0.454 $eÅ^{-3}$

3. Computational Section

DFT calculations were performed at the ωB97X-D/def2-TZVPP//B97-D/def2-SVP level of theory.[S10-S13] Stationary points on the potential energy surface (PES) were characterized by harmonic vibrational frequency calculations. Electronic structure analysis was carried out at the same level of theory as the geometry optimization. All calculations were carried out using GAUSSIAN 09 program.[S14]

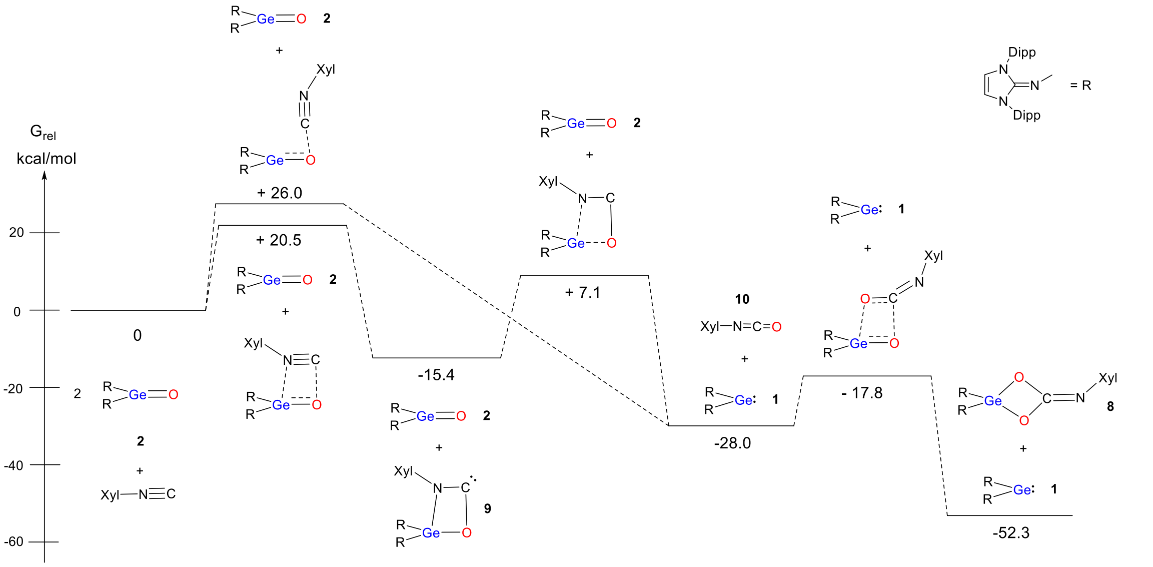

Figure S34. DFT-derived mechanism of the reaction of **2** and XylNC.

Table S2. NBO-Analysis of the central Ge in **2**.

2	Occupation	Atom	Polarization	*s*-character	*p*-character	*d*-character
Bond	1.93	Ge	22.59%	37.15%	62.34%	0.50%
		O	77.41%	19.00%	80.93%	0.08%
Bond	1.90	Ge	20.92%	31.40%	67.81%	0.79%
		N	79.08%	18.95%	81.01%	0.04%
Bond	1.90	Ge	20.92%	31.40%	67.81%	0.79%
		N	79.08%	18.95%	81.01%	0.04%

Table S3. Calculated Ge=O, Ge-N bond lengths [Å], NPA charges of Ge and O atoms, Wiberg Bond Index (WBI) and Mayer Bond Order (MBO) in **2**.

Compound	Bond length [Å]		NPA charge		WBI//MBO	
	Ge–O	Ge-N	Ge	O	Ge-O	Ge-N
**2**	1.670	1.808	+1.89	-1.02	1.30//1.75	0.76//1.03

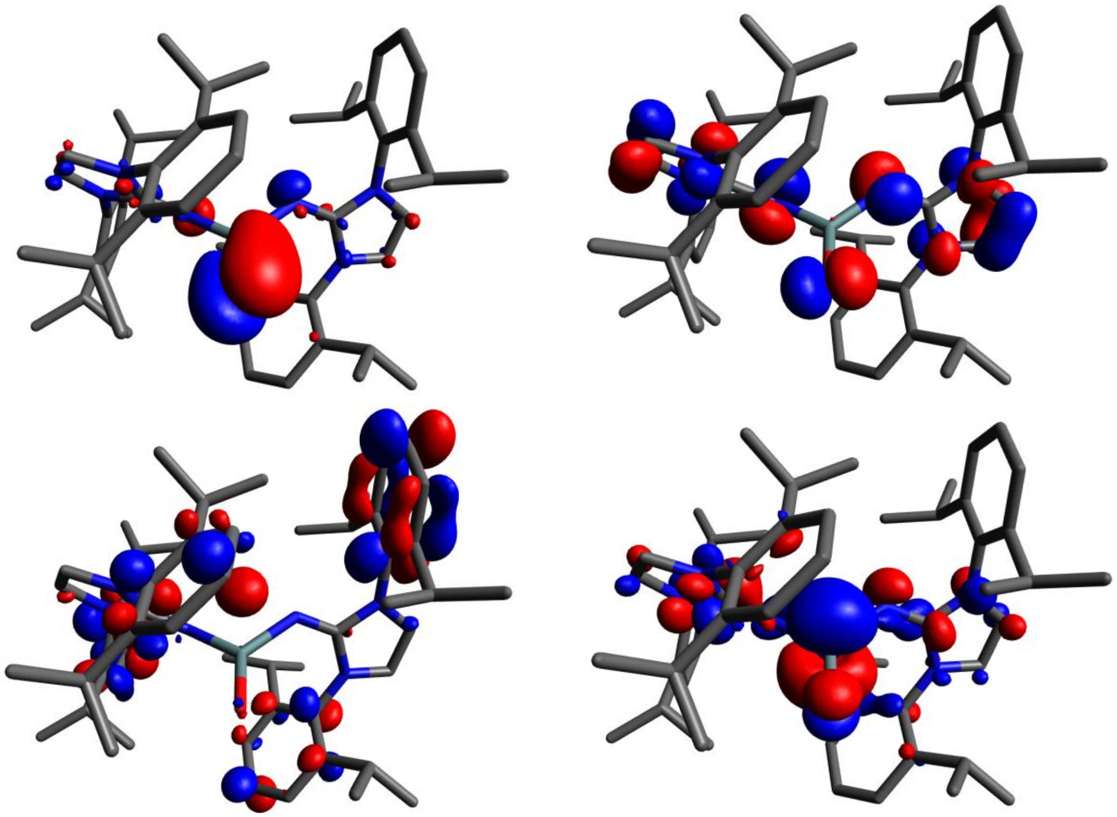

Figure S35. HOMO-3 (top left, -5.25 eV), HOMO (top right, –4.24 eV), LUMO (bottom left, –1.15 eV), and LUMO+9 (bottom right, –0.266 eV) of **2**.

Table S4. Cartesian coordinates of XylNC in Å.

Atomtype	X Coordinate [Å]	Y Coordinate [Å]	Z Coordinate [Å]
C	1.246100	-0.240301	0.000000
C	0.000000	0.441018	0.000000
C	-1.246099	-0.240301	0.000000
C	-1.217775	-1.646492	0.000000
C	0.000000	-2.344903	0.000000
C	1.217775	-1.646491	0.000000
N	0.000000	1.827038	0.000000
C	0.000003	3.015154	0.000000
C	-2.537743	0.539967	0.000000
H	-2.167176	-2.197283	0.000000
H	0.000001	-3.442216	0.000000
H	2.167175	-2.197284	0.000000
C	2.537742	0.539969	0.000000
H	-3.412645	-0.132335	0.000000
H	-2.601678	1.201617	0.885196
H	-2.601678	1.201617	-0.885196
H	3.412645	-0.132332	0.000000
H	2.601676	1.201619	-0.885196
H	2.601676	1.201619	0.885196

Table S5. Cartesian coordinates of **2** in Å.

Atomtype	X Coordinate [Å]	Y Coordinate [Å]	Z Coordinate [Å]
Ge	-0.000106	0.001422	-0.939312
O	-0.000129	0.003686	-2.609495
N	1.447517	0.177812	0.128748
N	2.299415	2.445959	-0.258881
N	3.592444	0.990213	0.780327
N	-1.447689	-0.177963	0.128311
N	-3.592511	-0.992426	0.777705
N	-2.299141	-2.445264	-0.265143
C	2.339843	1.113664	0.176532
C	3.498816	3.095730	0.057362
H	3.662552	4.138583	-0.210068
C	4.297127	2.197177	0.701092
H	5.299395	2.294388	1.116624
C	1.206844	3.000128	-1.002796
C	0.027015	3.373794	-0.301034
C	-1.038030	3.893158	-1.060261
H	-1.970092	4.178464	-0.562911
C	-0.931203	4.031630	-2.452394
H	-1.779483	4.429789	-3.023936
C	0.234089	3.637042	-3.119507
H	0.291244	3.722206	-4.210871
C	1.323857	3.094972	-2.411963
C	-0.053512	3.239142	1.220525
H	0.487734	2.315805	1.495978
C	-1.487747	3.081573	1.750331
H	-1.463737	2.862385	2.832346
H	-2.009004	2.248450	1.249816
H	-2.082302	4.005242	1.621860
C	0.660954	4.426719	1.904994
H	0.648842	4.301646	3.004725
H	0.148559	5.377388	1.662053
H	1.713905	4.511974	1.582303
C	2.574278	2.620337	-3.148487
H	3.184104	2.043356	-2.429819
C	2.242484	1.670065	-4.317486
H	1.589451	0.851798	-3.964628
H	3.178566	1.250456	-4.734130
H	1.728567	2.204358	-5.139321
C	3.421270	3.824224	-3.615304
H	3.711987	4.473162	-2.767902
H	2.853384	4.444026	-4.335437
H	4.344652	3.478358	-4.117693
C	4.088014	-0.246987	1.305281

C	3.952325	-0.500960	2.693795
C	4.422889	-1.735949	3.177883
H	4.329692	-1.972361	4.243896
C	4.993863	-2.679529	2.310754
H	5.344675	-3.641244	2.707268
C	5.111206	-2.405071	0.941962
H	5.552182	-3.154392	0.273154
C	4.665254	-1.179223	0.408798
C	3.244184	0.502541	3.603308
H	3.366374	1.503177	3.148370
C	1.727353	0.203926	3.643845
H	1.539631	-0.765353	4.142942
H	1.300389	0.146039	2.628272
H	1.191372	0.988008	4.210903
C	3.833987	0.572058	5.023861
H	3.369512	1.404012	5.585203
H	4.927802	0.733596	5.004140
H	3.636048	-0.354224	5.595676
C	4.771428	-0.894858	-1.088440
H	4.485026	0.159343	-1.252536
C	6.213741	-1.061559	-1.609375
H	6.923581	-0.431178	-1.041872
H	6.270052	-0.773655	-2.676057
H	6.554821	-2.111192	-1.531346
C	3.776520	-1.764786	-1.885227
H	4.022223	-2.839074	-1.781725
H	3.808948	-1.506777	-2.960331
H	2.744638	-1.611730	-1.529913
C	-2.339870	-1.114078	0.173624
C	-4.296927	-2.199342	0.695409
H	-5.299172	-2.297828	1.110696
C	-3.498427	-3.096078	0.049384
H	-3.661928	-4.138290	-0.220670
C	-4.088385	0.243337	1.305747
C	-4.666062	1.177576	0.411634
C	-5.112316	2.401976	0.947866
H	-5.553645	3.152787	0.280967
C	-4.994859	2.673116	2.317310
H	-5.345920	3.633744	2.716232
C	-4.423482	1.727586	3.182045
H	-4.330208	1.961412	4.248621
C	-3.952594	0.493939	2.694861
C	-4.772486	0.896792	-1.086264
H	-4.485796	-0.156923	-1.252960
C	-6.214970	1.064282	-1.606475
H	-6.924483	0.432321	-1.040324
H	-6.271427	0.778910	-2.673830

H	-6.556368	2.113619	-1.525863
C	-3.778033	1.768929	-1.881197
H	-4.024032	2.842891	-1.775055
H	-3.810642	1.513494	-2.956908
H	-2.746019	1.615327	-1.526503
C	-3.243962	-0.511561	3.601778
H	-3.365876	-1.511108	3.144374
C	-3.833504	-0.584830	5.022251
H	-3.368681	-1.418051	5.581419
H	-4.927274	-0.746655	5.002308
H	-3.635753	0.340067	5.596370
C	-1.727230	-0.212510	3.642799
H	-1.539768	0.755613	4.144229
H	-1.300452	-0.151980	2.627302
H	-1.190876	-0.997787	4.207849
C	-1.206374	-2.997342	-1.010329
C	-1.323118	-3.088434	-2.419768
C	-0.233127	-3.628414	-3.128567
H	-0.290063	-3.710640	-4.220167
C	0.932108	-4.024609	-2.462307
H	1.780554	-4.421099	-3.034763
C	1.038652	-3.889876	-1.069787
H	1.970658	-4.176396	-0.573027
C	-0.026608	-3.372711	-0.309361
C	-2.573457	-2.611978	-3.155253
H	-3.183530	-2.037060	-2.435142
C	-3.420154	-3.814713	-3.625558
H	-3.710938	-4.466029	-2.780006
H	-2.852012	-4.432430	-4.347278
H	-4.343491	-3.467619	-4.127182
C	-2.241559	-1.658459	-4.321577
H	-1.588730	-0.841064	-3.966330
H	-3.177612	-1.237847	-4.737270
H	-1.727390	-2.190428	-5.144763
C	0.053640	-3.242232	1.212575
H	-0.487658	-2.319664	1.490489
C	1.487790	-3.086124	1.743048
H	1.463610	-2.870037	2.825682
H	2.009088	-2.251552	1.244999
H	2.082396	-4.009400	1.612018
C	-0.660926	-4.431700	1.893646
H	-0.648985	-4.309670	2.993721
H	-0.148490	-5.381690	1.648156
H	-1.713828	-4.516068	1.570558

Table S6. Cartesian coordinates of transition state (+20.5 kcal/mol) in **Figure S34** in Å.

Atomtype	X Coordinate [Å]	Y Coordinate [Å]	Z Coordinate [Å]
C	3.689761	-3.420462	-2.044970
C	2.664797	-2.866531	-1.221499
C	2.490140	-3.305272	0.123367
C	3.347061	-4.301279	0.624017
C	4.364007	-4.850379	-0.173182
C	4.528863	-4.408166	-1.494840
N	1.851118	-1.832949	-1.666986
C	1.455375	-1.283705	-2.699818
O	0.433094	0.104119	-2.375297
Ge	-0.123342	0.500039	-0.767345
N	-1.553514	-0.218067	0.087971
C	-2.721178	-0.642349	-0.289898
N	-3.277306	-0.906777	-1.552910
C	-4.602570	-1.327641	-1.423868
C	-4.902941	-1.353120	-0.096079
N	-3.761674	-0.950503	0.601501
C	-2.534831	-1.017071	-2.780142
C	-1.980376	-2.277895	-3.113714
C	-1.302902	-2.380262	-4.341613
C	-1.181176	-1.272586	-5.191095
C	-1.732815	-0.037131	-4.831680
C	-2.415807	0.119190	-3.612313
C	-3.711372	-0.857661	2.033957
C	-4.348653	0.240427	2.659733
C	-4.406223	0.253931	4.068163
C	-3.830496	-0.776701	4.818606
C	-3.172875	-1.836373	4.175851
C	-3.097323	-1.904748	2.772214
C	-2.136257	-3.490663	-2.196730
C	-3.245322	-4.429983	-2.720733
C	-3.035841	1.454718	-3.207963
C	-2.131906	2.657055	-3.534249
C	-4.974114	1.374464	1.850232
C	-4.445951	2.753431	2.292964
C	-2.389294	-3.059152	2.060024
C	-1.351999	-3.781717	2.938585
C	1.448753	-2.657863	0.997825
C	3.876118	-2.945254	-3.464034
N	1.059495	0.825941	0.549232
C	1.970097	1.698399	0.824273
N	2.254056	2.963883	0.295345
C	3.271729	3.580007	1.040909
C	3.656636	2.709865	2.015552
N	2.883245	1.555829	1.879245
C	1.559695	3.607678	-0.782512

C	1.922259	3.293967	-2.119600
C	1.280277	4.013145	-3.147115
C	0.330720	5.000714	-2.855213
C	-0.016535	5.285231	-1.527566
C	0.588154	4.593526	-0.460156
C	2.996643	2.250029	-2.425675
C	4.410861	2.839653	-2.215079
C	0.178534	4.849986	0.991465
C	-0.873024	3.811461	1.441813
C	2.958968	0.372358	2.686352
C	1.914122	0.112109	3.605711
C	1.972840	-1.093517	4.333557
C	3.034498	-1.989225	4.158830
C	4.070588	-1.695907	3.258866
C	4.057418	-0.510327	2.500609
C	0.769046	1.094290	3.840601
C	0.829195	1.669803	5.272796
C	5.150729	-0.190116	1.478514
C	6.505651	-0.850285	1.792965
C	-0.602452	0.459941	3.545419
C	4.699168	-0.549161	0.044905
C	-0.334814	6.279105	1.249736
C	2.876518	1.637023	-3.831482
C	-0.821664	-4.265177	-1.983857
C	-4.437014	1.616153	-3.837798
C	-6.515796	1.318763	1.914967
C	-3.396300	-4.086359	1.491614
H	-5.816275	-1.628584	0.428839
H	-5.196171	-1.587651	-2.299320
H	-4.904858	1.085921	4.580164
H	-3.882261	-0.751599	5.914815
H	-2.712831	-2.622995	4.782743
H	-4.677398	1.236178	0.795396
H	-4.873827	3.550973	1.656562
H	-3.347070	2.795085	2.213715
H	-4.723270	2.982453	3.338756
H	-6.962720	2.112362	1.286660
H	-6.870484	1.464564	2.953106
H	-6.897563	0.343931	1.560446
H	-1.838195	-2.611770	1.212459
H	-0.650189	-3.070654	3.408853
H	-0.762109	-4.479547	2.318519
H	-1.836961	-4.376529	3.736083
H	-4.121237	-3.625561	0.800683
H	-3.962037	-4.566651	2.312788
H	-2.858960	-4.877877	0.936592
H	-1.608691	0.823699	-5.498265

H	-0.631960	-1.370199	-6.136078
H	-0.852731	-3.336249	-4.632532
H	-3.169684	1.438109	-2.109396
H	-2.059626	2.832370	-4.624333
H	-1.111393	2.501768	-3.144497
H	-2.540163	3.579589	-3.081248
H	-4.923114	2.545219	-3.482720
H	-5.093772	0.764110	-3.583281
H	-4.360734	1.668010	-4.940794
H	-2.458562	-3.126953	-1.206012
H	-4.197045	-3.888459	-2.870433
H	-3.426765	-5.254794	-2.005481
H	-2.951367	-4.873835	-3.691032
H	-0.481860	-4.757329	-2.914049
H	-0.969871	-5.058332	-1.226799
H	-0.013601	-3.600468	-1.642514
H	4.409887	2.798963	2.797469
H	3.618894	4.582250	0.793403
H	4.891273	-2.409582	3.135257
H	3.058651	-2.927443	4.728612
H	1.171730	-1.328787	5.045211
H	5.313562	0.903069	1.503568
H	6.828978	-0.652957	2.832057
H	7.280641	-0.459780	1.107687
H	6.465687	-1.945698	1.644990
H	5.374882	-0.092488	-0.702196
H	3.670841	-0.213114	-0.166872
H	4.717368	-1.642705	-0.092986
H	0.896246	1.941055	3.145988
H	0.038120	2.431055	5.414152
H	1.807717	2.143217	5.478484
H	0.671269	0.877130	6.028699
H	-0.834334	-0.345170	4.265531
H	-0.645175	0.047438	2.523736
H	-1.404556	1.215441	3.637419
H	1.522915	3.794424	-4.191801
H	-0.156581	5.545789	-3.673807
H	-0.770077	6.053570	-1.322695
H	2.862071	1.424000	-1.705324
H	4.560446	3.210365	-1.186150
H	4.588071	3.678830	-2.914914
H	5.175909	2.064473	-2.409688
H	1.846414	1.296180	-4.023114
H	3.530375	0.749751	-3.902503
H	3.195004	2.350681	-4.616359
H	1.074269	4.709398	1.625168
H	-1.130170	3.955144	2.508372

H	-0.518192	2.775773	1.315439
H	-1.797088	3.927975	0.844817
H	-0.463523	6.440871	2.335926
H	-1.320254	6.453334	0.778212
H	0.367171	7.042715	0.866917
H	3.221106	-4.635353	1.662284
H	5.029059	-5.622701	0.234998
H	5.325328	-4.835111	-2.119384
H	1.387949	-3.150921	1.980381
H	1.680469	-1.589977	1.161467
H	0.450622	-2.676223	0.525752
H	3.011604	-3.226467	-4.095998
H	3.925177	-1.840678	-3.513053
H	4.793472	-3.366050	-3.913049

Table S7. Cartesian coordinates of intermediate (-15.4 kcal/mol) in **Figure S34** in Å.

Atomtype	X Coordinate [Å]	Y Coordinate [Å]	Z Coordinate [Å]
C	-2.743531	-1.882633	2.960178
C	-2.353738	-0.535580	3.182426
C	-1.365829	-0.172789	4.131890
C	-0.755164	-1.204566	4.870705
C	-1.119482	-2.541167	4.669162
C	-2.102619	-2.874647	3.726996
N	-2.928501	0.509338	2.381776
C	-2.270204	1.042881	1.265922
N	-3.029553	2.169659	0.918253
C	-4.063258	2.357887	1.838199
C	-3.998435	1.340542	2.745453
C	-2.742795	2.928973	-0.264448
C	-1.572555	3.727301	-0.292748
C	-1.249445	4.368231	-1.505886
C	-2.058677	4.220299	-2.638746
C	-3.223747	3.440503	-2.579441
C	-3.592512	2.776882	-1.393757
C	-0.689823	3.932248	0.936285
C	0.778992	3.559355	0.666229
C	-4.823735	1.870067	-1.331274
C	-4.419160	0.393850	-1.534224
N	-1.158804	0.718338	0.711314
Ge	-0.209561	-0.622644	0.042168
O	-0.620157	-2.509541	0.439356
C	-0.937844	1.278888	4.335894
C	-1.030581	1.709958	5.813963
C	-3.842058	-2.230472	1.954212
C	-3.777002	-3.676277	1.431954

N	1.479828	0.021866	-0.027294
C	2.607034	-0.404868	-0.499880
N	3.059973	-1.674870	-0.902669
C	4.401164	-1.605446	-1.300872
C	4.803334	-0.311527	-1.186767
N	3.723936	0.424535	-0.689230
C	2.402337	-2.924540	-0.626834
C	1.796454	-3.641696	-1.690688
C	1.221229	-4.888648	-1.383490
C	1.232409	-5.390570	-0.075475
C	1.827212	-4.658262	0.956631
C	2.437407	-3.415970	0.703236
C	3.821771	1.820909	-0.361594
C	4.114124	2.198044	0.977381
C	4.365638	3.560800	1.225594
C	4.322644	4.508633	0.192395
C	3.985391	4.117493	-1.107387
C	3.711086	2.767672	-1.407537
C	1.744536	-3.080315	-3.113028
C	0.493720	-3.527629	-3.896118
C	3.117618	-2.655133	1.840545
C	4.248918	-3.489846	2.477637
C	4.147219	1.159801	2.102905
C	5.445399	0.319912	2.088560
C	3.316365	2.356802	-2.824062
C	4.544579	2.334035	-3.759501
C	3.010387	-3.446185	-3.922724
C	2.104408	-2.183610	2.903870
C	3.958578	1.770407	3.503344
C	2.202536	3.258884	-3.394533
C	0.475176	1.514694	3.767711
C	-5.239309	-1.959083	2.561181
C	-5.934257	2.259730	-2.323749
C	-0.818788	5.380573	1.458623
H	5.762703	0.161625	-1.390092
H	4.946994	-2.498877	-1.598294
H	4.598891	3.894571	2.241840
H	4.534702	5.563326	0.410560
H	3.927582	4.870063	-1.902668
H	3.290546	0.482346	1.925034
H	3.794791	0.964690	4.241732
H	3.091375	2.449475	3.541315
H	4.855278	2.333165	3.825986
H	5.425763	-0.421185	2.909318
H	6.326666	0.972693	2.237354
H	5.579624	-0.232291	1.144779
H	2.912158	1.329882	-2.773040

H	1.334301	3.298490	-2.714129
H	1.861265	2.868646	-4.371119
H	2.559438	4.292386	-3.561901
H	5.320120	1.639386	-3.389528
H	4.997381	3.341338	-3.833534
H	4.254223	2.012979	-4.777862
H	1.817727	-5.053917	1.979729
H	0.756211	-6.355330	0.141023
H	0.729413	-5.466715	-2.172135
H	3.592681	-1.754576	1.418614
H	1.587993	-3.038191	3.377043
H	1.329992	-1.533849	2.465358
H	2.617516	-1.609909	3.698987
H	4.783195	-2.894030	3.241867
H	4.983453	-3.813139	1.716909
H	3.853365	-4.395196	2.974959
H	1.708753	-1.978022	-3.017887
H	3.931651	-3.039630	-3.474784
H	2.933299	-3.049709	-4.953110
H	3.115979	-4.546007	-3.988020
H	0.592515	-4.573566	-4.243919
H	0.358733	-2.892203	-4.789649
H	-0.414960	-3.452921	-3.280750
H	-4.747062	3.202718	1.762537
H	-4.593833	1.141708	3.635286
H	-3.844865	3.335781	-3.475149
H	-1.782443	4.717368	-3.577918
H	-0.347398	4.989389	-1.559469
H	-5.253169	1.959854	-0.316515
H	-6.200831	3.330034	-2.243839
H	-6.841576	1.659611	-2.125607
H	-5.634058	2.055467	-3.368554
H	-4.062747	0.234976	-2.565285
H	-5.283092	-0.275197	-1.361162
H	-3.605292	0.089442	-0.855110
H	-1.052608	3.262518	1.733395
H	-0.215770	5.512424	2.377472
H	-1.869498	5.635299	1.693397
H	-0.453835	6.108251	0.708736
H	1.241031	4.225351	-0.085254
H	0.869004	2.517524	0.317609
H	1.373685	3.658398	1.591934
H	-2.359931	-3.927255	3.574200
H	-0.625105	-3.334835	5.244298
H	0.025444	-0.957315	5.600432
H	-3.714712	-1.553497	1.087192
H	-5.360481	-0.909620	2.876604

H	-5.403953	-2.604911	3.444859
H	-6.029895	-2.183975	1.820349
H	-2.787143	-3.907655	1.004975
H	-4.534344	-3.816476	0.639372
H	-4.011913	-4.404821	2.231508
H	-1.630240	1.925966	3.769750
H	0.759945	2.577253	3.881777
H	0.523200	1.259913	2.695449
H	1.219896	0.901460	4.306726
H	-0.794309	2.786340	5.913429
H	-0.312910	1.154232	6.446329
H	-2.044648	1.538008	6.220181
C	-1.207756	-2.751199	-0.713626
N	-1.169204	-1.610667	-1.446079
C	-1.788023	-1.327227	-2.684995
C	-1.360136	-0.173745	-3.409829
C	-1.982500	0.148377	-4.630204
C	-3.006524	-0.644623	-5.161409
C	-3.424268	-1.772656	-4.445102
C	-2.852109	-2.132192	-3.208740
C	-0.271908	0.738322	-2.895658
H	-1.646572	1.044300	-5.169025
H	-3.477913	-0.384205	-6.117602
H	-4.238016	-2.394233	-4.841991
C	-3.424270	-3.326948	-2.482245
H	-0.033776	1.512022	-3.642005
H	-0.574368	1.268311	-1.971946
H	0.660312	0.190665	-2.667825
H	-2.698190	-4.155524	-2.409179
H	-3.669820	-3.077652	-1.435034
H	-4.337766	-3.687082	-2.991087

Table S8. Cartesian coordinates of transition state (+7.1 kcal/mol) in **Figure S34** in Å.

Atomtype	X Coordinate [Å]	Y Coordinate [Å]	Z Coordinate [Å]
C	2.852759	2.843245	-2.721088
C	1.788390	1.958023	-2.350124
C	1.397638	0.916509	-3.244304
C	2.073355	0.772165	-4.470711
C	3.108798	1.636620	-4.845327
C	3.480323	2.663959	-3.969156
N	1.133545	2.060102	-1.101801
C	1.095999	3.085535	-0.262045
O	0.616744	2.865142	0.875945
Ge	0.191741	0.710870	0.329820
N	-1.441578	-0.061030	-0.027533

C	-2.584286	0.377238	-0.443685
N	-3.105864	1.674538	-0.641991
C	-4.429048	1.602469	-1.094073
C	-4.755980	0.289128	-1.220885
N	-3.650988	-0.463744	-0.814776
C	-2.528011	2.903362	-0.169525
C	-2.001137	3.830166	-1.107250
C	-1.530301	5.059112	-0.608238
C	-1.564325	5.347690	0.762227
C	-2.074897	4.411624	1.667098
C	-2.577514	3.174751	1.222572
C	-3.686019	-1.898999	-0.756020
C	-4.001757	-2.533876	0.476222
C	-4.206605	-3.926893	0.452860
C	-4.095985	-4.658995	-0.738747
C	-3.736822	-4.016641	-1.927919
C	-3.508303	-2.625972	-1.957458
C	-1.936094	3.518912	-2.604040
C	-3.214164	3.983807	-3.341019
C	-3.190551	2.196937	2.224946
C	-2.177920	1.740717	3.294588
C	-4.099910	-1.730719	1.777177
C	-3.950566	-2.596305	3.041966
C	-3.092972	-1.940924	-3.258166
C	-1.927825	-2.678365	-3.949554
C	0.297108	-0.058542	-2.907573
C	3.345882	3.952824	-1.823068
N	1.238236	-0.751964	0.501919
C	2.272369	-1.224797	1.097594
N	2.803326	-0.985802	2.374198
C	3.848044	-1.887167	2.633902
C	4.021395	-2.655969	1.520622
N	3.083895	-2.241772	0.572202
C	2.222053	-0.073082	3.315491
C	2.619969	1.291353	3.295410
C	1.985056	2.162660	4.201604
C	1.006299	1.695834	5.090092
C	0.639717	0.344347	5.098138
C	1.240458	-0.569752	4.211084
C	3.714467	1.777556	2.341944
C	5.114426	1.323590	2.820910
C	0.801981	-2.032706	4.171567
C	-0.458836	-2.187250	3.296002
C	2.885542	-2.748939	-0.753849
C	1.770926	-3.586878	-0.998696
C	1.535878	-3.987124	-2.329663
C	2.379749	-3.571175	-3.367180

C	3.489490	-2.755916	-3.095080
C	3.767300	-2.326631	-1.783473
C	0.851171	-4.066942	0.120766
C	0.962023	-5.596467	0.303443
C	4.928237	-1.381053	-1.471679
C	6.124683	-1.527334	-2.429470
C	-0.606766	-3.630433	-0.109579
C	4.432436	0.081264	-1.447093
C	0.582151	-2.638335	5.571686
C	3.726445	3.301081	2.126002
C	-0.701400	4.131044	-3.298235
C	-4.457499	2.797657	2.872027
C	-5.413526	-0.920691	1.876762
C	-4.295266	-1.789119	-4.215155
H	-5.679998	-0.192375	-1.537107
H	-5.020982	2.502430	-1.249649
H	-4.456717	-4.455651	1.378245
H	-4.272991	-5.742349	-0.731813
H	-3.624876	-4.602019	-2.848339
H	-3.252099	-1.018836	1.762085
H	-3.835476	-1.946074	3.928237
H	-3.070403	-3.257791	2.989038
H	-4.846430	-3.223867	3.210705
H	-5.433899	-0.348368	2.823014
H	-6.286105	-1.601635	1.870724
H	-5.531200	-0.201311	1.051019
H	-2.734509	-0.927709	-3.002439
H	-1.072388	-2.804328	-3.263970
H	-1.585331	-2.103866	-4.829976
H	-2.233793	-3.677831	-4.311404
H	-5.109443	-1.203993	-3.751021
H	-4.702235	-2.781161	-4.489906
H	-3.990719	-1.273905	-5.145945
H	-2.086122	4.641609	2.739541
H	-1.173029	6.305837	1.127213
H	-1.109842	5.796090	-1.300395
H	-3.513030	1.296635	1.677665
H	-1.814595	2.590617	3.900817
H	-1.295045	1.259150	2.841073
H	-2.647431	1.011475	3.981959
H	-4.931914	2.061910	3.548748
H	-5.197929	3.085576	2.102777
H	-4.217379	3.699150	3.466544
H	-1.868919	2.418595	-2.700791
H	-4.123040	3.483450	-2.968812
H	-3.130731	3.770107	-4.423714
H	-3.348495	5.075651	-3.218816

H	-0.829972	5.218541	-3.458566
H	-0.555014	3.666069	-4.289803
H	0.214772	3.976104	-2.709021
H	4.724139	-3.461760	1.311056
H	4.356956	-1.899363	3.596733
H	4.137881	-2.439276	-3.918902
H	2.173937	-3.885031	-4.399007
H	0.676695	-4.631904	-2.551353
H	5.292269	-1.626467	-0.456568
H	6.455367	-2.578988	-2.517327
H	6.975607	-0.924324	-2.061837
H	5.880684	-1.156412	-3.442546
H	4.130312	0.397921	-2.459324
H	5.232250	0.760705	-1.096308
H	3.555364	0.205425	-0.790992
H	1.188460	-3.600318	1.062195
H	0.334063	-5.928083	1.152444
H	2.005356	-5.907233	0.500773
H	0.614229	-6.132332	-0.600330
H	-1.033433	-4.102855	-1.012670
H	-0.688275	-2.535836	-0.212627
H	-1.239063	-3.936412	0.742980
H	2.244658	3.225578	4.206448
H	0.517480	2.397549	5.778284
H	-0.134536	-0.001822	5.792341
H	3.520626	1.300533	1.361469
H	5.196641	0.228676	2.911365
H	5.343119	1.770545	3.807358
H	5.888189	1.660691	2.105350
H	2.737700	3.677264	1.819066
H	4.458050	3.553204	1.337070
H	4.045583	3.833242	3.042643
H	1.608704	-2.615417	3.691102
H	-0.753892	-3.251057	3.233084
H	-0.294018	-1.816284	2.271143
H	-1.301264	-1.620828	3.732776
H	0.396503	-3.725481	5.487641
H	-0.297880	-2.197480	6.076748
H	1.461776	-2.485073	6.224015
H	1.769201	-0.041323	-5.142653
H	3.621105	1.512143	-5.807819
H	4.294722	3.346490	-4.246786
H	0.006075	-0.634540	-3.799888
H	0.616794	-0.784607	-2.135882
H	-0.602265	0.447640	-2.517803
H	2.590253	4.751375	-1.704642
H	3.545873	3.591314	-0.798943

H	4.272238	4.399862	-2.228578

Table S9. Cartesian coordinates of **1** in Å.

Atomtype	X Coordinate [Å]	Y Coordinate [Å]	Z Coordinate [Å]
C	-0.112632	-3.480924	-0.229350
C	-1.260114	-3.111545	-0.985391
C	-1.349271	-3.325565	-2.383705
C	-0.278337	-3.987406	-3.017203
C	0.852596	-4.378837	-2.291027
C	0.938828	-4.116076	-0.914832
N	-2.345553	-2.465682	-0.315609
C	-2.336414	-1.099523	0.054557
N	-3.610787	-0.909635	0.621016
C	-4.353896	-2.095159	0.582724
C	-3.575651	-3.053004	0.004166
C	-4.041675	0.323489	1.201589
C	-4.586962	1.323850	0.360988
C	-4.996055	2.532335	0.959574
C	-4.869461	2.729350	2.340656
C	-4.321985	1.724800	3.152898
C	-3.891855	0.503474	2.601853
N	-1.392058	-0.236695	-0.033366
Ge	-0.000286	0.001582	-1.248779
N	1.391023	0.237637	-0.032370
C	2.335547	1.100101	0.057341
N	2.345228	2.466799	-0.310836
C	3.575284	3.053358	0.010460
C	4.353011	2.094464	0.587979
N	3.609590	0.909071	0.624213
C	1.260856	3.113208	-0.981794
C	0.112075	3.481680	-0.227299
C	-0.938693	4.116620	-0.914049
C	-0.850499	4.380138	-2.289976
C	0.281837	3.989837	-3.014592
C	1.352140	3.328196	-2.379825
C	4.040619	-0.325497	1.201606
C	3.893523	-0.508010	2.601857
C	4.323892	-1.730684	3.149703
C	4.868918	-2.734133	2.334422
C	4.992873	-2.534588	0.953478
C	4.583498	-1.324645	0.358018
C	0.043433	3.193313	1.272070
C	0.845393	4.247147	2.068930
C	2.546388	2.832465	-3.192769
C	3.400317	4.009124	-3.709939

C	3.208473	0.558499	3.456382
C	3.799923	0.692270	4.871513
C	4.691222	-1.124609	-1.151947
C	3.692470	-2.037989	-1.892796
C	-4.696599	1.126856	-1.149241
C	-3.696795	2.040059	-1.888973
C	-3.204294	-0.563967	3.453145
C	-1.682238	-0.296571	3.507936
C	-2.542026	-2.828741	-3.198162
C	-2.097048	-1.902801	-4.350178
C	-0.046068	-3.193352	1.270268
C	-0.849577	-4.247296	2.065416
C	-1.389966	3.071185	1.810756
C	2.103718	1.905900	-4.345180
C	1.685732	0.294686	3.509771
C	6.132733	-1.324801	-1.662259
C	-6.138091	1.330445	-1.658158
C	-3.796000	-0.703561	4.867642
C	-3.396448	-4.004665	-3.716201
C	1.386601	-3.072030	1.811115
H	3.776890	4.101594	-0.207426
H	5.366663	2.138464	0.985283
H	-1.847322	4.398527	-0.372838
H	-1.684432	4.876566	-2.802942
H	0.328465	4.178093	-4.094324
H	0.520402	2.210369	1.431146
H	-1.364619	2.745139	2.865130
H	-1.968129	2.321668	1.244677
H	-1.931088	4.035702	1.784278
H	0.838772	3.997650	3.147338
H	0.395870	5.251487	1.946553
H	1.898041	4.298378	1.738262
H	3.183345	2.230420	-2.520240
H	1.481517	1.075357	-3.964231
H	2.988715	1.478614	-4.854142
H	1.514490	2.454333	-5.104286
H	3.758564	4.641396	-2.876482
H	2.814234	4.652218	-4.394011
H	4.282269	3.637227	-4.265363
H	4.218359	-1.910420	4.225667
H	5.190621	-3.684117	2.780884
H	5.410168	-3.330915	0.324519
H	3.349721	1.530617	2.949378
H	1.472619	-0.625900	4.084837
H	1.264932	0.166391	2.498009
H	1.162759	1.133669	4.005950
H	3.348236	1.559139	5.388956

H	4.896041	0.836666	4.843610
H	3.588780	-0.200175	5.490569
H	4.404793	-0.080499	-1.371488
H	6.841705	-0.658233	-1.136604
H	6.191071	-1.107676	-2.745730
H	6.473590	-2.366852	-1.513610
H	3.942926	-3.105420	-1.740969
H	3.710728	-1.835983	-2.980785
H	2.665994	-1.873096	-1.524808
H	-5.367726	-2.139984	0.979482
H	-3.776888	-4.100956	-0.215412
H	-5.415008	3.329657	0.332984
H	-5.190907	3.678276	2.789547
H	-4.214231	1.902569	4.228979
H	-4.412080	0.082677	-1.371030
H	-6.847958	0.664636	-1.132751
H	-6.197654	1.114679	-2.741830
H	-6.476916	2.372962	-1.508184
H	-3.944787	3.107561	-1.733684
H	-3.716988	1.841176	-2.977509
H	-2.670189	1.871877	-1.522790
H	-3.342943	-1.534818	2.943056
H	-3.342416	-1.570924	5.382610
H	-4.891747	-0.850563	4.838823
H	-3.587306	0.187476	5.489549
H	-1.471545	0.623028	4.085501
H	-1.261248	-0.164896	2.496675
H	-1.157646	-1.135616	4.002273
H	-0.323380	-4.174981	-4.097118
H	1.687051	-4.875436	-2.802975
H	1.846535	-4.398678	-0.372431
H	-3.179164	-2.225961	-2.526450
H	-3.756370	-4.636428	-2.883086
H	-2.810063	-4.648434	-4.399379
H	-4.277328	-3.632031	-4.272834
H	-1.474305	-1.072954	-3.968595
H	-2.981000	-1.474518	-4.860109
H	-1.507683	-2.451978	-5.108640
H	-0.522889	-2.210313	1.429160
H	1.359767	-2.746747	2.865693
H	1.965853	-2.322312	1.246420
H	1.927463	-4.036694	1.784758
H	-0.844450	-3.998342	3.143959
H	-0.400236	-5.251736	1.943195
H	-1.901751	-4.297960	1.733162

Table S10. Cartesian coordinates of XylNCO in Å.

Atomtype	X Coordinate [Å]	Y Coordinate [Å]	Z Coordinate [Å]
C	1.044490	1.118249	0.000295
C	-0.005879	0.162204	0.001213
C	0.257325	-1.235915	0.000766
C	1.600529	-1.655187	-0.000834
C	2.651976	-0.726033	-0.001882
C	2.370181	0.648805	-0.001315
N	-1.314413	0.643211	0.003148
C	-2.496829	0.384429	-0.000896
C	-0.880216	-2.228682	0.001957
H	1.819238	-2.731142	-0.001239
H	3.692436	-1.074391	-0.003101
H	3.191144	1.377749	-0.002104
C	0.720762	2.593571	0.000966
H	-0.507110	-3.266988	0.002248
H	-1.529887	-2.100846	0.890371
H	-1.530980	-2.101891	-0.885823
H	1.638434	3.206913	0.000418
H	0.115074	2.868349	-0.883999
H	0.116418	2.867848	0.886996
O	-3.672239	0.260409	-0.003428

Table S11. Cartesian coordinates of transition state (-17.8 kcal/mol) in **Figure S34** in Å.

Atomtype	X Coordinate [Å]	Y Coordinate [Å]	Z Coordinate [Å]
C	5.437682	-2.312524	0.888437
C	4.080206	-2.729307	0.779035
C	3.711763	-3.794428	-0.090018
C	4.720557	-4.385896	-0.876047
C	6.057599	-3.967236	-0.797049
C	6.410062	-2.940142	0.091415
N	3.155636	-2.069552	1.584871
C	2.185526	-1.31009	1.482896
O	1.408035	-0.560385	2.052955
Ge	0.142031	-0.261673	-0.059524
O	1.489577	-1.281386	-0.36523
C	2.285249	-4.279199	-0.167247
C	5.791402	-1.172536	1.813371
N	0.142753	1.497236	-0.357599
C	0.868185	2.218518	-1.16111
N	1.339761	1.965579	-2.455391
C	1.972349	3.103632	-2.966
C	1.918185	4.072185	-2.008414
N	1.252555	3.53189	-0.903321

C	1.343907	0.666277	-3.068193
C	2.561002	-0.054866	-3.113062
C	2.525968	-1.355768	-3.65142
C	1.326939	-1.913586	-4.105901
C	0.134899	-1.174521	-4.059123
C	0.116088	0.136937	-3.548633
C	3.876825	0.523881	-2.597795
C	4.490983	-0.349997	-1.491435
C	-1.15077	0.993767	-3.55292
C	-1.093838	2.034567	-4.694863
C	0.986322	4.21944	0.328229
C	-0.020111	5.219454	0.332934
C	-0.246497	5.906265	1.541224
C	0.483838	5.588391	2.694255
C	1.449404	4.572242	2.66893
C	1.72736	3.860726	1.48498
C	-0.876195	5.46747	-0.912211
C	-1.996438	4.404395	-1.019392
C	2.786356	2.755076	1.436154
C	3.105775	2.136278	2.807768
N	-1.503015	-0.863524	0.152975
C	-2.371877	-1.363631	0.961679
N	-2.446387	-1.389346	2.359832
C	-3.636161	-2.012116	2.764979
C	-4.317664	-2.378827	1.645364
N	-3.552297	-1.985029	0.543762
C	-1.490677	-0.820135	3.268683
C	-0.681476	-1.696871	4.035708
C	0.228746	-1.115262	4.937193
C	0.336741	0.276392	5.051812
C	-0.475188	1.120163	4.282979
C	-1.421797	0.591833	3.38418
C	-4.004922	-2.013359	-0.815375
C	-3.556224	-3.047505	-1.670182
C	-4.069989	-3.074979	-2.982613
C	-4.990622	-2.112405	-3.416885
C	-5.391444	-1.077098	-2.559307
C	-4.895595	-0.994633	-1.24493
C	-0.760833	-3.211356	3.83371
C	-0.295488	-4.029941	5.051382
C	-2.384266	1.491321	2.606043
C	-3.78379	1.492087	3.264207
C	-2.527431	-4.079165	-1.214374
C	-1.193328	-3.891921	-1.968372
C	-5.211646	0.203904	-0.348843
C	-6.687945	0.639112	-0.398097

C	0.024457	-3.631527	2.572059
C	-1.878855	2.92849	2.422494
C	-3.057489	-5.521562	-1.351076
C	-4.26769	1.380653	-0.692709
C	-1.462977	6.887587	-0.997214
C	4.079747	3.248379	0.747477
C	-2.445578	0.174743	-3.630313
C	4.86637	0.754586	-3.760009
H	-3.875128	-2.128302	3.821188
H	-5.278931	-2.874659	1.516387
H	-0.369125	2.204923	4.384487
H	1.07073	0.709761	5.743387
H	0.880759	-1.754381	5.541488
H	-2.489684	1.066701	1.593276
H	-2.572083	3.483076	1.767614
H	-0.883716	2.946654	1.950768
H	-1.829051	3.478715	3.380231
H	-4.486246	2.104615	2.66713
H	-3.732291	1.923971	4.281948
H	-4.20603	0.475226	3.343537
H	-1.821215	-3.472288	3.659214
H	-0.284133	-3.063034	1.678682
H	-0.124554	-4.708145	2.364498
H	1.104009	-3.454832	2.716768
H	-0.804496	-3.71043	5.979586
H	0.795486	-3.940052	5.209468
H	-0.511582	-5.10197	4.888218
H	-6.079676	-0.307495	-2.927657
H	-5.382732	-2.15426	-4.441292
H	-3.740224	-3.861772	-3.672203
H	-4.995235	-0.084473	0.694934
H	-4.488979	1.775182	-1.702077
H	-3.210728	1.064161	-0.672463
H	-4.398693	2.205337	0.032198
H	-6.872195	1.435911	0.346532
H	-7.366724	-0.205423	-0.17729
H	-6.963865	1.04862	-1.388044
H	-2.32484	-3.902258	-0.143189
H	-4.005098	-5.657163	-0.797047
H	-2.317097	-6.240632	-0.952918
H	-3.242795	-5.786407	-2.409323
H	-1.324179	-4.062157	-3.05425
H	-0.437223	-4.609045	-1.599825
H	-0.792255	-2.875104	-1.823796
H	2.293081	5.094675	-1.998347
H	2.419992	3.099915	-3.958763
H	1.991832	4.326865	3.587701

H	0.287658	6.129417	3.629106
H	-1.01358	6.686556	1.589637
H	2.367884	1.938552	0.823249
H	3.88589	3.630632	-0.270226
H	4.805302	2.417348	0.664067
H	4.549954	4.059066	1.336418
H	3.636968	2.848793	3.468077
H	3.76099	1.257969	2.671409
H	2.189942	1.784559	3.311618
H	-0.22567	5.336559	-1.796435
H	-1.952367	7.034556	-1.977748
H	-0.681709	7.661168	-0.879799
H	-2.233535	7.059313	-0.222138
H	-2.755407	4.568568	-0.232327
H	-1.606037	3.380054	-0.894575
H	-2.503509	4.474931	-2.000436
H	3.447614	-1.948947	-3.678797
H	1.313827	-2.940757	-4.492511
H	-0.792207	-1.633061	-4.416185
H	3.670037	1.508251	-2.14317
H	4.438623	1.420765	-4.533327
H	5.1309	-0.201782	-4.249313
H	5.801663	1.212637	-3.386029
H	3.763964	-0.48892	-0.677394
H	5.405896	0.123772	-1.090014
H	4.769822	-1.354435	-1.855432
H	-1.180412	1.546298	-2.596127
H	-3.319361	0.839275	-3.524171
H	-2.49712	-0.578286	-2.826532
H	-2.551283	-0.343993	-4.601237
H	-1.985865	2.689195	-4.663609
H	-1.073174	1.528225	-5.678856
H	-0.198027	2.676255	-4.620607
H	4.443771	-5.200423	-1.559459
H	6.823885	-4.447561	-1.41927
H	7.454858	-2.609057	0.1646
H	6.883082	-1.01185	1.859967
H	5.318094	-0.231475	1.469856
H	5.40992	-1.351857	2.836187
H	2.187623	-5.113443	-0.884801
H	1.93794	-4.633663	0.820803
H	1.614966	-3.454465	-0.468822

Table S12. Cartesian coordinates of **8** in **Figure S34** in Å.

Atomtype	X Coordinate [Å]	Y Coordinate [Å]	Z Coordinate [Å]
C	-0.668694	-1.522222	3.917608
C	-1.514142	-0.665109	3.170289
C	-1.470451	0.750091	3.289955
C	-0.544952	1.294104	4.200189
C	0.277302	0.464688	4.975962
C	0.213204	-0.925928	4.838356
N	-2.469342	-1.2481	2.265089
C	-2.399217	-1.248933	0.864881
N	-3.605752	-1.825903	0.462125
C	-4.385069	-2.164087	1.572519
C	-3.684211	-1.809513	2.683054
N	-1.517996	-0.797974	0.02581
Ge	0.127034	-0.147167	0.150768
N	0.270482	1.531388	-0.37785
C	1.046062	2.181062	-1.18958
N	1.563952	1.843527	-2.444263
C	2.257275	2.934649	-2.98184
C	2.199328	3.951824	-2.075695
N	1.468642	3.489871	-0.976597
C	1.560937	0.507677	-2.974667
C	2.766898	-0.234732	-2.947055
C	2.718996	-1.569435	-3.394764
C	1.517572	-2.139916	-3.826723
C	0.338818	-1.378832	-3.856428
C	0.334365	-0.033402	-3.444337
C	1.214843	4.201399	0.243571
C	1.906516	3.799449	1.416352
C	1.623263	4.506319	2.601456
C	0.701858	5.563273	2.611179
C	0.025677	5.930296	1.439351
C	0.256964	5.246796	0.229829
C	4.087672	0.360064	-2.4602
C	4.721322	-0.461964	-1.324487
C	-0.913062	0.845284	-3.550511
C	-2.224791	0.053029	-3.623311
C	2.941651	2.671492	1.380819
C	4.292626	3.19258	0.840178
C	-0.546839	5.546697	-1.038186
C	-1.714195	4.541749	-1.186312
C	-4.100944	-1.800507	-0.884492
C	-4.85267	-0.670201	-1.296892
C	-5.384161	-0.683126	-2.599976
C	-5.156628	-1.766018	-3.460809
C	-4.381402	-2.854283	-3.038777
C	-3.838824	-2.896963	-1.738938

C	-5.004818	0.558536	-0.400755
C	-6.453088	1.078783	-0.3254
C	-2.945053	-4.052964	-1.299743
C	-3.577802	-5.432736	-1.569061
C	-2.425491	1.641714	2.493056
C	-1.884766	3.057844	2.244042
C	-0.693022	-3.039245	3.735734
C	-1.568941	-3.71449	4.816233
O	1.132599	-0.569202	1.695599
C	1.923854	-1.392649	0.968804
O	1.519991	-1.33058	-0.332161
N	2.878536	-2.07114	1.479179
C	5.06702	0.536648	-3.641211
C	-0.790479	1.80652	-4.755878
C	3.121741	1.941355	2.722824
C	-1.058862	6.995066	-1.127558
C	-4.029191	1.668547	-0.853902
C	-1.556171	-3.927158	-1.963782
C	-3.814359	1.700946	3.168856
C	0.716425	-3.667782	3.734635
H	2.740543	2.86848	-3.955395
H	2.609716	4.960381	-2.100769
H	-0.58971	-1.844533	-4.199658
H	1.493595	-3.192219	-4.137611
H	3.629626	-2.178622	-3.365863
H	-0.964527	1.460237	-2.633946
H	-3.082688	0.745187	-3.591799
H	-2.325082	-0.64087	-2.772299
H	-2.310661	-0.523466	-4.563172
H	-1.668506	2.478921	-4.801626
H	-0.745725	1.235237	-5.702828
H	0.115844	2.434492	-4.69158
H	3.886698	1.363409	-2.04599
H	4.035061	-0.536554	-0.467833
H	5.656776	0.018939	-0.982653
H	4.969803	-1.492591	-1.631954
H	4.62969	1.161426	-4.442823
H	5.327455	-0.442874	-4.084663
H	6.004742	1.015029	-3.300183
H	-0.704338	6.746253	1.473133
H	0.500112	6.101025	3.546783
H	2.129428	4.225712	3.531184
H	0.123085	5.38923	-1.90356
H	-2.483023	4.734339	-0.415741
H	-1.37776	3.498302	-1.063593
H	-2.193025	4.647109	-2.178269
H	-1.509281	7.175078	-2.12113

H	-0.243549	7.726777	-0.977959
H	-1.843494	7.198894	-0.374749
H	2.574758	1.911365	0.672261
H	4.180451	3.648621	-0.1603
H	5.016782	2.360164	0.758357
H	4.717294	3.95536	1.520426
H	3.613492	2.581784	3.480233
H	3.757102	1.049827	2.575501
H	2.153531	1.587325	3.115009
H	-5.367695	-2.618653	1.453502
H	-3.9284	-1.89106	3.740222
H	-4.18752	-3.682696	-3.730676
H	-5.572311	-1.752474	-4.476641
H	-5.964967	0.176867	-2.954236
H	-2.7968	-3.963816	-0.208003
H	-4.577925	-5.516795	-1.10454
H	-2.935182	-6.232726	-1.156246
H	-3.689079	-5.627577	-2.652434
H	-1.643407	-3.995982	-3.065182
H	-0.886825	-4.737853	-1.622567
H	-1.085924	-2.960912	-1.714526
H	-4.714329	0.272959	0.625187
H	-6.520427	1.905118	0.407027
H	-7.152824	0.281696	-0.012733
H	-6.799325	1.474115	-1.298833
H	-4.294948	2.035988	-1.863082
H	-2.991785	1.294435	-0.883719
H	-4.069992	2.526105	-0.157819
H	0.882543	-1.559691	5.430281
H	0.990716	0.911371	5.680301
H	-0.461619	2.380573	4.304776
H	-1.151138	-3.242851	2.748775
H	-2.610574	-3.349526	4.8018
H	-1.154237	-3.513028	5.822429
H	-1.589156	-4.811005	4.66871
H	1.413935	-3.120853	3.078712
H	0.657334	-4.718415	3.394001
H	1.149794	-3.682756	4.752753
H	-2.560375	1.180631	1.499953
H	-2.571759	3.602819	1.574206
H	-0.893014	3.033045	1.763517
H	-1.81051	3.646462	3.176738
H	-4.514294	2.298392	2.553801
H	-3.739373	2.176387	4.165412
H	-4.252082	0.69604	3.299691
C	3.705058	-2.956493	0.781407
C	5.11075	-2.809793	0.990093

C	6.001834	-3.662919	0.318721
C	5.530361	-4.67704	-0.53118
C	4.148166	-4.8554	-0.685232
C	3.21663	-4.020531	-0.03445
C	5.611028	-1.724091	1.91465
H	7.082286	-3.530826	0.469595
H	6.235796	-5.339768	-1.049353
H	3.771441	-5.671698	-1.318027
C	1.735721	-4.276715	-0.196491
H	1.546053	-5.320794	-0.506483
H	1.190673	-4.089984	0.747331
H	1.294019	-3.59964	-0.949198
H	6.704422	-1.789757	2.060767
H	5.374656	-0.717216	1.519442
H	5.10782	-1.785704	2.898053

Table S13. Cartesian coordinates of transition state (26.0 kcal/mol) in **Figure S34** in Å.

Atomtype	X Coordinate [Å]	Y Coordinate [Å]	Z Coordinate [Å]
C	4.178517	3.696842	-0.380802
C	3.219506	2.762032	-0.859488
C	3.135178	2.416323	-2.242146
C	4.05433	3.014307	-3.124409
C	5.0115	3.933064	-2.665708
C	5.066818	4.27413	-1.304607
N	2.372595	2.189117	0.083631
C	1.397379	1.468623	0.14662
O	0.800312	0.887413	1.941782
Ge	0.027426	0.121504	0.560185
N	-1.640385	0.34147	-0.090072
C	-2.529099	1.280355	-0.075985
N	-2.599185	2.545034	0.538637
C	-3.822617	3.162146	0.249372
C	-4.532757	2.323311	-0.553406
N	-3.757036	1.17945	-0.752858
C	-1.550787	3.195956	1.273864
C	-0.782615	4.193251	0.62103
C	0.209778	4.847821	1.375841
C	0.429857	4.511964	2.717973
C	-0.33729	3.515471	3.335351
C	-1.343083	2.834987	2.627175
C	-4.148193	0.072077	-1.573543
C	-5.070391	-0.869946	-1.046325
C	-5.471244	-1.929236	-1.882286
C	-4.973451	-2.043916	-3.189336
C	-4.064224	-1.101841	-3.683522

C	-3.631042	-0.022982	-2.887198
C	-1.043343	4.595527	-0.830844
C	-1.830234	5.923817	-0.895302
C	-2.207547	1.761764	3.286191
C	-1.463631	0.95777	4.364517
C	-5.543277	-0.763003	0.403714
C	-4.499269	-1.414031	1.338049
C	-2.638634	0.993921	-3.44615
C	-1.267509	0.343346	-3.721506
C	2.092587	1.448756	-2.739887
C	4.214889	4.039999	1.089437
N	0.561788	-1.483462	-0.038439
C	1.435708	-2.3694	0.285105
N	2.12589	-3.159438	-0.655599
C	2.88183	-4.142254	-0.015301
C	2.726732	-3.965239	1.327292
N	1.875262	-2.868473	1.521876
C	2.084833	-2.860093	-2.053701
C	3.218635	-2.245647	-2.655586
C	3.110374	-1.871992	-4.008452
C	1.922091	-2.086591	-4.725282
C	0.827562	-2.706706	-4.111092
C	0.886403	-3.115723	-2.763611
C	4.488806	-1.9786	-1.839213
C	5.76441	-1.855771	-2.691923
C	-0.292738	-3.852011	-2.128764
C	-0.491709	-5.227029	-2.805028
C	1.348268	-2.486812	2.80071
C	2.029862	-1.504146	3.564376
C	1.509172	-1.200788	4.835902
C	0.350571	-1.829827	5.313251
C	-0.316268	-2.780289	4.529771
C	0.175108	-3.137307	3.258342
C	3.304341	-0.844232	3.040249
C	4.542259	-1.707401	3.377087
C	-0.558099	-4.165035	2.396247
C	-1.820325	-3.539077	1.768944
C	0.246283	4.694205	-1.667341
C	-3.507758	2.385755	3.841291
C	-6.944478	-1.34941	0.650436
C	-3.195757	1.695583	-4.703045
C	3.505647	0.598553	3.536285
C	-0.894254	-5.455998	3.169812
C	-1.589154	-3.021585	-2.153171
C	4.340435	-0.740474	-0.923761
H	-5.521654	2.420124	-1.000168
H	-4.062307	4.145191	0.650861

H	-6.178894	-2.679034	-1.511089
H	-5.295384	-2.879653	-3.82417
H	-3.676246	-1.205931	-4.704349
H	-5.590068	0.311161	0.662303
H	-4.774701	-1.264227	2.399736
H	-3.491237	-0.998978	1.169262
H	-4.446271	-2.501245	1.145278
H	-7.276032	-1.112386	1.678434
H	-6.950462	-2.451731	0.555536
H	-7.689613	-0.941464	-0.057551
H	-2.484585	1.774162	-2.679692
H	-0.838451	-0.080621	-2.798755
H	-0.561903	1.091416	-4.127093
H	-1.352106	-0.471654	-4.463891
H	-4.172768	2.171456	-4.497523
H	-3.33596	0.981486	-5.536576
H	-2.494688	2.478858	-5.04919
H	-0.133912	3.246766	4.377033
H	1.216997	5.024579	3.286281
H	0.822621	5.625079	0.903883
H	-2.497808	1.043204	2.4988
H	-1.268713	1.563938	5.270425
H	-0.502525	0.588315	3.969294
H	-2.073959	0.090799	4.678237
H	-4.154434	1.603815	4.283995
H	-4.083062	2.899626	3.049505
H	-3.274066	3.125675	4.630796
H	-1.671803	3.810821	-1.291187
H	-2.788393	5.858469	-0.350004
H	-2.049718	6.19795	-1.944857
H	-1.240209	6.743423	-0.442752
H	0.887786	5.533701	-1.341466
H	-0.000218	4.862789	-2.73242
H	0.841408	3.772902	-1.589674
H	3.447766	-4.885065	-0.576631
H	3.108116	-4.536888	2.172125
H	3.95787	-1.392836	-4.510196
H	1.85477	-1.771329	-5.77486
H	-0.091256	-2.880755	-4.684344
H	4.633656	-2.84932	-1.173782
H	5.890048	-2.718993	-3.371367
H	6.651281	-1.799525	-2.034019
H	5.757721	-0.934219	-3.304309
H	4.392462	0.190042	-1.514935
H	5.157853	-0.71174	-0.179419
H	3.382662	-0.73891	-0.379278
H	-0.045271	-4.040264	-1.070325

H	-1.304062	-5.787802	-2.304433
H	0.429904	-5.837505	-2.760899
H	-0.769795	-5.111721	-3.87016
H	-1.913916	-2.809417	-3.187479
H	-1.459585	-2.064903	-1.62115
H	-2.408879	-3.577998	-1.661807
H	2.002436	-0.443616	5.454225
H	-0.047218	-1.56044	6.300148
H	-1.230811	-3.252408	4.908564
H	3.209396	-0.798937	1.94132
H	4.463397	-2.726381	2.960274
H	4.666269	-1.794049	4.473856
H	5.458597	-1.242419	2.965264
H	2.60713	1.19996	3.324171
H	4.367877	1.049917	3.009878
H	3.735995	0.631956	4.61886
H	0.111362	-4.45378	1.566317
H	-2.346287	-4.273992	1.130032
H	-1.562494	-2.668559	1.142378
H	-2.521276	-3.207458	2.557184
H	-1.330058	-6.207483	2.484723
H	-1.635231	-5.273751	3.970676
H	0.008239	-5.893342	3.635948
H	5.8131	4.996853	-0.949522
H	5.716123	4.388099	-3.373658
H	4.009827	2.755938	-4.190685
H	3.233156	4.419282	1.42923
H	4.413295	3.138793	1.699455
H	4.989106	4.796089	1.308084
H	2.154608	0.469244	-2.233231
H	1.071118	1.820351	-2.538494
H	2.188369	1.273577	-3.824468

4. References

[S1] D. Franz, E. Irran, S. Inoue, *Dalton Trans.* **2014**, *43*, 4451-4461.

[S2] J. Kouvetakis, A. Haaland, D. J. Shorokhov, H. V. Volden, G. V. Girichev, V. I. Sokolov, P. Matsunaga, *J. Am. Chem. Soc.* **1998**, *120*, 6738-6744.

[S3] *APEX suite of crystallographic software*, APEX 3 version 2015.5-2; Bruker AXS Inc.: Madison, Wisconsin, USA, 2015.

[S4] *SAINT*, Version 7.56a and SADABS Version 2008/1; Bruker AXS Inc.: Madison, Wisconsin, USA, 2008.

[S5] G. M. Sheldrick, *SHELXL-2014*, University of Göttingen, Göttingen, Germany, 2014.

[S6] C. B. Hübschle, G. M. Sheldrick, B. Dittrich, *J. Appl. Cryst.* **2011**, *44*, 1281-1284.

[S7] G. M. Sheldrick, *SHELXL-97*, University of Göttingen, Göttingen, Germany, 1998.

[S8] A. J. C. Wilson, *International Tables for Crystallography*, Vol. C, Tables 6.1.1.4 (pp. 500-502), 4.2.6.8 (pp. 219-222), and 4.2.4.2 (pp. 193-199); Kluwer Academic Publishers: Dordrecht, The Netherlands, 1992.

[S9] C. F. Macrae, I. J. Bruno, J. A. Chisholm, P. R. Edgington, P. McCabe, E. Pidcock, L. Rodriguez-Monge, R. Taylor, J. van de Streek, P. A. Wood, *J. Appl. Cryst.* **2008**, *41*, 466-470.

[S10] F. Weigend, R. Ahlrichs, *Phys. Chem. Chem. Phys.* **2005**, *7*, 3297-3305.

[S11] A. D. Becke, *J. Chem. Phys*. **1997**, *107*, 8554-8560.

[S12] S. Grimme, *J. Comput. Chem.* **2006**, *27*, 1787-1799.

[S13] J.-D. Chai, M. Head-Gordon, *Phys. Chem. Chem. Phys.* **2008**, *10*, 6615-6620.

[S14] Gaussian 09, Revision B.01, M. J. Frisch, G. W. Trucks, H. B. Schlegel, G. E. Scuseria, M. A. Robb, J. R. Cheeseman, G. Scalmani, V. Barone, B. Mennucci, G. A. Petersson, H. Nakatsuji, M. Caricato, X. Li, H. P. Hratchian, A. F. Izmaylov, J. Bloino, G. Zheng, J. L. Sonnenberg, M. Hada, M. Ehara, K. Toyota, R. Fukuda, J. Hasegawa, M. Ishida, T. Nakajima, Y. Honda, O. Kitao, H. Nakai, T. Vreven, J. A. Montgomery, Jr., J. E. Peralta, F. Ogliaro, M. Bearpark, J. J. Heyd, E. Brothers, K. N. Kudin, V. N. Staroverov, R. Kobayashi, J. Normand, K. Raghavachari, A. Rendell, J. C. Burant, S. S. Iyengar, J. Tomasi, M. Cossi, N. Rega, J. M. Millam, M. Klene, J. E. Knox, J. B. Cross, V. Bakken, C. Adamo, J. Jaramillo, R. Gomperts, R. E. Stratmann, O. Yazyev, A. J. Austin, R. Cammi, C. Pomelli, J. W. Ochterski, R. L. Martin, K. Morokuma, V. G. Zakrzewski, G. A. Voth, P. Salvador, J. J. Dannenberg, S. Dapprich, A. D. Daniels, Ö. Farkas, J. B. Foresman, J. V. Ortiz, J. Cioslowski, and D. J. Fox, Gaussian, Inc., Wallingford CT, 2009.
